# The Clinical Implications of Sex on Waitlist Outcomes in Patients With Acute-on-Chronic Liver Failure

**DOI:** 10.1016/j.gastha.2026.100970

**Published:** 2026-04-13

**Authors:** Youngjae Cha, Ki Jung Lee, Mohammed Rifat Shaik, Gregory Fan, Martin Banks, Sarah Yang, Lauren Thompson, Andrew Chan, Janet Liang, Nishat Anjum Shaik, Sharmitha Yerneni, Mohamed Refaat, David Uihwan Lee

**Affiliations:** 1 Division of Gastroenterology and Hepatology, University of Maryland, Baltimore, Maryland; 2 Department of Medicine, Tufts University School of Medicine, Boston, Massachusetts

**Keywords:** Acute-on-Chronic Liver Failure, Waitlisting, Sex Disparity, Liver Transplantation

## Abstract

**Background and Aims:**

Previous studies have examined the role of sex in the development and prognosis of acute-on-chronic liver failure (ACLF). To our knowledge, few studies have investigated sex-specific waitlist mortality rates stratified by ACLF grade. This question is significant, as women have historically faced greater barriers to liver transplantation and higher waitlist mortality rates compared to men with chronic liver disease. Therefore, understanding whether these disparities persist in ACLF—a condition with intermediate to high acuity—is essential for optimizing patient care and outcomes.

**Methods:**

The study population was identified using United Network for Organ Sharing transplant data from 2005 to 2019 and included adult patients registered for liver transplantation. Candidates were stratified by ACLF grade at the time of waitlist registration and further stratified by sex. The primary outcomes were waitlist mortality and liver transplantation, analyzed using a competing-risk regression model to account for transplantation as a competing event. Secondary outcomes included cause-specific mortality, which was evaluated using cause-specific Cox proportional hazards models.

**Results:**

In the fully adjusted competing-risk regression model, male sex was associated with lower waitlist mortality among candidates without ACLF (subdistribution hazard ratio [SHR]: 0.87; 95% confidence interval [CI]: 0.83–0.90), ACLF grade 1 (SHR: 0.83; 95% CI: 0.73–0.95), and ACLF grade 2 (SHR: 0.81; 95% CI: 0.70–0.95), while no statistically significant difference was observed in ACLF grade 3 (SHR: 0.92; 95% CI: 0.80–1.06). Male sex was associated with higher liver transplantation rates across all ACLF strata.

**Conclusion:**

Competing risk analysis demonstrated higher waitlist mortality among women than men in ACLF grades 1 and 2, potentially reflecting delayed transplant prioritization among female candidates.

## Introduction

Acute-on-chronic liver failure (ACLF) is characterized by a severe systemic inflammatory response and the failure of one or more organ systems in individuals with pre-existing chronic liver disease.^1^^,^^2^ Excessive alcohol consumption is the leading cause of chronic liver disease, with alcohol and infection being the most common precipitants for the development of ACLF.^3^ ACLF has been increasingly recognized as a growing entity, now with an estimated incidence rate of 20.1 cases per 1000 person-years.^4^ Several diagnostic criteria for ACLF have been established by organizations such as the Asian Pacific Association for the Study of the Liver, the European Association for the Study of the Liver-Chronic Liver Failure Consortium (EASL-CLIF), and the North American Consortium for the Study of End-Stage Liver Disease, although some variation exists across these definitions.^5^, ^6^, ^7^ EASL-CLIF criteria, which are widely used, define ACLF as the failure of one of 6 major organ systems in patients with acutely decompensated cirrhosis.^8^ Grading is based on the number of organ failures and is classified into grades 1, 2, and 3.

The 28-day transplant-free mortality, in fact, correlates with ACLF grade, with rates as high as 77% in ACLF grade 3.^9^ Prognosis is closely tied to hepatic reserve, immune function, and systemic inflammation, and treatment typically involves intensive care to address precipitating events and organ failure.^10^, ^11^, ^12^ Bridging therapies such as plasma exchange can provide temporary relief; however, liver transplantation (LT) remains the only definitive treatment that substantially improves survival.^13^^,^^14^ Access to LT, however, is limited by organ availability. Barriers at the recipient level include inequities in access, limited social networks, challenges related to immigration status, and a general lack of awareness about living donor LT. For donors, obstacles consist of worries about surgical risks, the time required for recovery, financial pressures, and religious considerations. At the provider level, barriers reflect gaps in institutional support and a shortage of specialized surgeons.^36^ There is some concern for futility of LT in ACLF grade 3 patients, especially with 4 or more organ failures, Chronic Liver Failure Consortium acute-on-chronic liver failure score > 64 or in those with other general contraindications for LT.

Previous studies have explored the role of sex in the development and prognosis of ACLF across various clinical settings. Data from the National Health and Nutrition Examination Survey revealed that females have an 8% higher risk of developing ACLF.^15^ The Predisposition, Infection, Response, Organ failure prediction model highlighted sex as a predictor of 90-day mortality in ACLF from hepatitis B, while Klein et al found female sex to be an independent risk factor for the development of ACLF following surgery in patients with cirrhosis.^16^^,^^17^ The underlying reasons for this association remain unclear, and sex is often excluded from key ACLF prognostic models, including the EASL-CLIF C model.^9^

To our knowledge, there are only a few studies that have investigated sex-specific transplantation rates and waitlist mortality rates stratified by ACLF grade. This question is particularly important, as women have historically encountered greater barriers to LT and higher waitlist mortality rates compared to men in the context of overall liver disease.^18^^,^^19^ Notably, this sex disparity in transplantation rates does not appear in cases of acute liver failure, where the urgency of the condition negates these differences.^20^ Therefore, understanding whether these disparities persist in ACLF—a condition with intermediate to high acuity—is important for optimizing patient care and outcomes.

## Methods

### Database

The United Network for Organ Sharing (UNOS)-Standard Transplant Analysis and Research registry is derived from data collected by the UNOS-Organ Procurement and Transplantation Network. All data in the UNOS-Standard Transplant Analysis and Research registry are de-identified to ensure compliance with data-user agreement protocols and adhere to the Health Resources and Services Administration Contract 234-2005-370011C to safeguard patient confidentiality. The registry includes a wide range of deidentified information, such as demographic, clinical, biomedical, laboratory, and socioeconomic variables. While our study utilizes data from the UNOS registry, the findings do not necessarily reflect the policies of the Department of Health and Human Services.

### Study Population and Variables

The study population was identified using UNOS transplantation data from 2005 to 2019. Patients under 18 years of age, those diagnosed with hepatocellular carcinoma, those registered for multi-organ transplantation, and those with acute liver failure and compensated cirrhosis were excluded from the analysis. To evaluate postlisting outcomes, all candidates were included regardless of if they got a final LT, consistent with appropriate modeling of pretransplant mortality. The primary exposure variable was sex, with the study cohort divided into male and female candidates. The primary endpoint was all-cause mortality. Covariates included demographic factors, liver disease etiology, medical comorbidities, immunosuppressant medications, hepatic complications, biomarkers, laboratory values, critical care rate, and life-support variables, as well as donor characteristics.

ACLF grading was assessed at the time of waitlist registration and was operationalized using a modified version of the EASL-CLIF criteria adapted for compatibility with the UNOS registry.^9^^,^^21^ Surrogates for organ failure included the need for dialysis, mechanical ventilation, international normalized ratio thresholds, total bilirubin levels, and hepatic encephalopathy grade. The EASL-CLIF definition of kidney failure was also incorporated, applying a serum creatinine threshold of ≥1.5 mg/dL to define renal dysfunction. ACLF grades 0–3 were assigned at waitlist registration using the EASL-CLIF criteria based on the number and type of organ failures. The study examined postlisting outcomes (transplantation rate and waitlist mortality) among liver transplant candidates who had ACLF at the time of waitlist registration and did not evaluate changes in ACLF status over time.

### Statistics

Baseline characteristics were summarized by sex within each ACLF grade. Standardized mean differences (SMDs) were used to quantify baseline balance, with SMD >0.10 interpreted as meaningful imbalance. *P* values were not used to assess baseline balance given their sensitivity to large sample sizes; narrative interpretation emphasized clinically meaningful differences based on SMDs.

**Figure 1:**
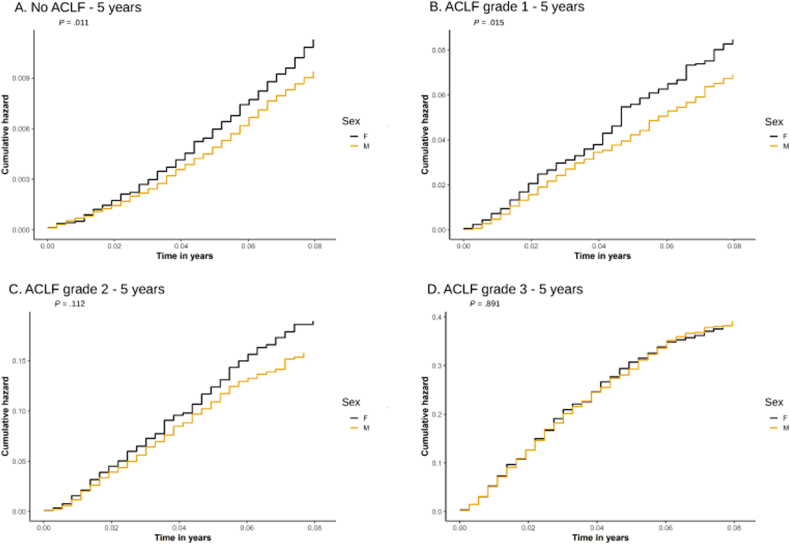
Cumulative hazard curves for all-cause mortality—5 years. (A–D) The 5-year cumulative hazard for all-cause mortality among patients on the liver transplant waitlist without acute-on-chronic liver failure (ACLF) and with ACLF grades 1–3, stratified by sex.

Study endpoints were compared using case-incidence rates. Waitlist outcomes were modeled using Fine–Gray subdistribution hazard models to account for the competing risk of transplantation.^22^ Time zero was defined as the date of waitlist registration, and follow-up continued until death, transplantation, waitlist removal, or administrative censoring, whichever occurred first; these results are listed in Table 1. Event-specific Cox proportional hazards models were used to estimate cause-specific hazards for waitlist mortality and transplantation; these results are presented in the Supplementary Tables. Figure 1 represents cumulative hazard curves that were produced and demonstrated the relationship between sex and waitlist mortality.

**Table 1:** Baseline Characteristics of Waitlisted Patients by Sex for Acute-on-Chronic Liver Failure Grade 1

Variable	Comparison of female vs male						
	Female			Male			SMD^a^
	n = 2965	35.19	%	n = 5460	64.81	%	
Recipient demographics							
Age (y)	54.30	±10.40	Unit	54.40	±9.15	Unit	0.008
Race							0.118^a^
White (%)	2085	70.32	%	4096	75.02	%	
Black (%)	293	9.88	%	396	7.25	%	
Hispanic (%)	459	15.48	%	777	14.23	%	
Asian (%)	79	2.66	%	120	2.20	%	
Other (%)	49	1.65	%	71	1.30	%	
BMI (kg/m^2^)	29.30	±6.68	Unit	29.30	±5.75	Unit	0.006
Comorbidities							
Hepatitis B (%)	43	1.45	%	204	3.74	%	0.144^a^
Hepatitis C (%)	712	24.01	%	1905	34.89	%	0.240^a^
Alcoholic liver disease (%)	963	32.48	%	2756	50.48	%	0.372^a^
Diabetes (%)	664	22.39	%	1227	22.47	%	0.002
Hepatic variables							
Ascites							0.099
Absent (%)	197	6.64	%	360	6.59	%	
Slight (%)	1548	52.21	%	2593	47.49	%	
Moderate (%)	1220	41.15	%	2507	45.92	%	
Encephalopathy							0.064
None (%)	344	11.60	%	695	12.73	%	
Grade 1–2 (%)	2494	84.11	%	4471	81.89	%	
Grade 3–4 (%)	127	4.28	%	294	5.38	%	
TIPS procedure (%)	234	7.89	%	450	8.24	%	0.013
MELD score (unit)	26.10	±5.12	Unit	26.00	±5.23	Unit	−0.008
Biomarkers							
Albumin (mg/dL)	3.04	±0.81	Unit	2.96	±0.75	Unit	−0.097
Serum creatinine (mg/dL)	1.77	±1.17	Unit	1.96	±1.30	Unit	0.155^a^
INR	2.14	±2.02	Unit	2.09	±0.81	Unit	−0.036
Bilirubin (mg/dL)	10.70	±9.83	Unit	9.36	±9.53	Unit	−0.135
Life support variables							
Artificial life support (%)	0	0.00	%	0	0.00	%	
Primary inotropic agent							0.101^a^
Dobutamine (%)	46	1.55	%	92	1.68	%	
Dopamine (%)	308	10.39	%	653	11.96	%	
Epinephrine (%)	16	0.54	%	26	0.48	%	
Levophed (%)	257	8.67	%	577	10.57	%	
Neosynephrine (%)	287	9.68	%	574	10.51	%	
None (%)	1998	67.39	%	3430	62.82	%	
Other (%)	53	1.79	%	108	1.98	%	
Ventilator support (%)	0	0.00	%	0	0.00	%	

INR, international normalized ratio; TIPS, transjugular intrahepatic portosystemic shunt.

a SMD >0.1.

**Table 2:** Baseline Characteristics of Waitlisted Patients Without Acute-on-Chronic Liver Failure by Sex

Variable	Comparison of female vs male						
	Female			Male			SMD
	n = 21,549	34.57	%	n = 40,790	65.43	%	
Recipient demographics							
Age (y)	55.30	±9.48	Unit	55.00	±8.39	Unit	−0.031
Race							0.091
White (%)	15,717	72.94	%	31,184	76.45	%	
Black (%)	1480	6.87	%	2246	5.51	%	
Hispanic (%)	3397	15.76	%	5841	14.32	%	
Asian (%)	599	2.78	%	1069	2.62	%	
Other (%)	356	1.65	%	450	1.10	%	
BMI (kg/m^2^)	29.00	±6.31	Unit	29.30	±5.34	Unit	0.053
Comorbidities							
Hepatitis B (%)	361	1.68	%	1270	3.11	%	0.094
Hepatitis C (%)	7637	35.44	%	18,563	45.51	%	0.206^a^
Alcoholic liver disease (%)	5374	24.94	%	17,164	42.08	%	0.369^a^
Diabetes (%)	5299	24.59	%	9381	23.00	%	0.037
Hepatic variables							
Ascites							0.077
Absent (%)	4337	20.13	%	8040	19.71	%	
Slight (%)	13,113	60.85	%	23,734	58.19	%	
Moderate (%)	4099	19.02	%	9016	22.10	%	
Encephalopathy							0.032
None (%)	7927	36.79	%	14,411	35.33	%	
Grade 1–2 (%)	12,876	59.75	%	25,012	61.32	%	
Grade 3–4 (%)	746	3.46	%	1367	3.35	%	
TIPS procedure (%)	1952	9.06	%	4148	10.17	%	0.038
MELD score (unit)	14.90	±4.74	Unit	15.20	±4.58	Unit	0.063
Biomarkers							
Albumin (mg/dL)	3.02	±0.62	Unit	2.98	±0.62	Unit	−0.069
Serum creatinine (mg/dL)	0.93	±0.32	Unit	1.03	±0.31	Unit	0.335^a^
INR	1.48	±0.36	Unit	1.48	±0.37	Unit	0.022
Bilirubin (mg/dL)	3.44	±3.57	Unit	3.25	±3.14	Unit	−0.056
Life support variables							
Artificial life support (%)	0	0.00	%	0	0.00	%	
Primary inotropic agent							0.071
Dobutamine (%)	170	0.79	%	372	0.91	%	
Dopamine (%)	1514	7.03	%	3344	8.20	%	
Epinephrine (%)	113	0.52	%	190	0.47	%	
Levophed (%)	1394	6.47	%	2853	6.99	%	
Neosynephrine (%)	1392	6.46	%	3040	7.45	%	
None (%)	16,768	77.81	%	30,587	74.99	%	
Other (%)	198	0.92	%	404	0.99	%	
Ventilator support (%)	0	0.00	%	0	0.00	%	

INR, international normalized ratio; TIPS, transjugular intrahepatic portosystemic shunt.

a SMD >0.1.

**Table 3:** Baseline Characteristics of Waitlisted Patients by Sex for Acute-on-Chronic Liver Failure Grade 2

Variable	Comparison of female vs male						
	Female			Male			SMD^a^
	n = 2251	35.70	%	n = 4054	64.30	%	
Recipient demographics							
Age (y)	52.10	±10.80	Unit	51.80	±9.78	Unit	0.029
Race							0.168^a^
White (%)	1485	65.97	%	2854	70.40	%	
Black (%)	271	12.04	%	305	7.52	%	
Hispanic (%)	367	16.30	%	660	16.28	%	
Asian (%)	75	3.33	%	168	4.14	%	
Other (%)	53	2.35	%	67	1.65	%	
BMI (kg/m^2^)	30.20	±7.16	Unit	29.70	±6.07	Unit	0.077
Comorbidities							
Hepatitis B (%)	63	2.80	%	207	5.11	%	0.119^a^
Hepatitis C (%)	495	21.99	%	1218	30.04	%	0.184^a^
Alcoholic liver disease (%)	879	39.05	%	2349	57.94	%	0.385^a^
Diabetes (%)	442	19.64	%	712	17.56	%	0.053
Hepatic variables							
Ascites							0.098
Absent (%)	209	9.28	%	330	8.14	%	
Slight (%)	996	44.25	%	1643	40.53	%	
Moderate (%)	1046	46.47	%	2081	51.33	%	
Encephalopathy							0.018
None (%)	455	20.21	%	790	19.49	%	
Grade 1–2 (%)	1345	59.75	%	2445	60.31	%	
Grade 3–4 (%)	451	20.04	%	819	20.20	%	
TIPS procedure (%)	172	7.64	%	303	7.47	%	0.006
MELD score (unit)	32.20	±5.76	Unit	33.00	±6.07	Unit	−0.124
Biomarkers							
Albumin (mg/dL)	3.08	±0.81	Unit	3.02	±0.79	Unit	0.071
Serum creatinine (mg/dL)	1.89	±1.33	Unit	2.19	±1.54	Unit	−0.205
INR	2.78	±1.13	Unit	2.70	±1.13	Unit	0.070
Bilirubin (mg/dL)	18.30	±11.80	Unit	19.20	±12.80	Unit	−0.070
Life support variables							
Artificial life support (%)	1	0.04	%	0	0.00	%	0.030
Primary inotropic agent							0.056
Dobutamine (%)	38	1.69	%	73	1.80	%	
Dopamine (%)	245	10.88	%	452	11.15	%	
Epinephrine (%)	11	0.49	%	29	0.72	%	
Levophed (%)	221	9.82	%	424	10.46	%	
Neosynephrine (%)	248	11.02	%	487	12.01	%	
None (%)	1446	64.24	%	2521	62.19	%	
Other (%)	42	1.87	%	68	1.68	%	
Ventilator support (%)	55	2.44	%	91	2.24	%	0.013

INR, international normalized ratio; TIPS, transjugular intrahepatic portosystemic shunt.

a SMD >0.1.

**Table 4:** Baseline Characteristics of Waitlisted Patients by Sex for Acute-on-Chronic Liver Failure Grade 3

Variable	Comparison of female vs male						
	Female			Male			SMD^a^
	n = 1847	38.22	%	n = 2985	61.78	%	
Recipient demographics							
Age (y)	51.60	±11.10	Unit	51.70	±9.93	Unit	0.012
Race							0.140^a^
White (%)	1190	64.43	%	1960	65.66	%	
Black (%)	240	12.99	%	282	9.45	%	
Hispanic (%)	304	16.46	%	540	18.09	%	
Asian (%)	70	3.79	%	154	5.16	%	
Other (%)	43	2.33	%	49	1.64	%	
BMI (kg/m^2^)	30.70	±7.53	Unit	30.20	±6.25	Unit	−0.078
Comorbidities							
Hepatitis B (%)	49	2.65	%	229	7.67	%	0.228^a^
Hepatitis C (%)	457	24.74	%	1020	34.17	%	0.208^a^
Alcoholic liver disease (%)	688	37.25	%	1669	55.91	%	0.381^a^
Diabetes (%)	311	16.84	%	564	18.89	%	0.054
Hepatic variables							
Ascites							0.080
Absent (%)	138	7.47	%	192	6.43	%	
Slight (%)	611	33.08	%	904	30.28	%	
Moderate (%)	1098	59.45	%	1889	63.28	%	
Encephalopathy							0.141^a^
None (%)	142	7.69	%	288	9.65	%	
Grade 1–2 (%)	609	32.97	%	1131	37.89	%	
Grade 3–4 (%)	1096	59.34	%	1566	52.46	%	
TIPS procedure (%)	131	7.09	%	237	7.94	%	0.032
MELD score	35.40	±7.49	Unit	37.90	±7.63	Unit	0.331^a^
Biomarkers							
Albumin (mg/dL)	3.21	±0.86	Unit	3.08	±0.77	Unit	−0.161
Serum creatinine (mg/dL)	2.18	±1.38	Unit	2.79	±1.67	Unit	0.398^a^
INR	3.08	±1.66	Unit	3.07	±1.48	Unit	−0.009
Bilirubin (mg/dL)	22.50	±12.70	Unit	24.50	±13.20	Unit	0.154^a^
Life support variables							
Artificial life support (%)	1	0.05	%	4	0.13	%	0.026
Primary inotropic agent							0.074
Dobutamine (%)	41	2.22	%	62	2.08	%	
Dopamine (%)	143	7.74	%	270	9.05	%	
Epinephrine (%)	11	0.60	%	16	0.54	%	
Levophed (%)	144	7.80	%	273	9.15	%	
Neosynephrine (%)	190	10.29	%	309	10.35	%	
None (%)	1279	69.25	%	2000	67.00	%	
Other (%)	39	2.11	%	55	1.84	%	
Ventilator support (%)	775	41.96	%	951	31.86	%	0.210^a^

INR, international normalized ratio; TIPS, transjugular intrahepatic portosystemic shunt.

a SMD >0.1.

Patterns of missing variable expressions were assessed graphically, and the random forest imputation was implemented under the assumption that data were missing at random and was applied only to variables with low missingness, consistent with established methodological standards.^23^ Variable-level missingness and the distribution of missingness are illustrated in Supplementary Figure 7, demonstrating that most variables had <1% missing data. Variables with >20% missingness were excluded from the analytic dataset to reduce potential bias associated with imputing nonessential, sparsely populated variables. A complete-case sensitivity analysis was considered but ultimately not completed, as it would have excluded a substantial proportion of observations and introduced selection bias, particularly by disproportionately omitting sicker or more complex patients. Given the minimal missingness among included variables, random forest imputation was selected to preserve analytic integrity and minimize bias. To further interrogate the independent and potentially heterogeneous contributions of body size components, we additionally performed stratified and adjusted analyses incorporating height and weight separately, which are presented in Supplementary Tables 3.1–3.6. All analyses were conducted in R (R version 3.6.3; RStudio version 1.2.5042).

The Supplementary Tables and Figures provide additional details for the unified cohort, including transplanted candidates. Supplementary Figures 1 and 2 present the 30-day and 90-day survival curves, respectively. Supplementary Figures 3–6 display the multivariable forest plots. Data analysis was performed using RStudio version 1.2.5042 and R version 3.6.3. All *P* values with a type I error (α) of <.05 were considered statistically significant.

## Results

### Patient Population and Baseline Characteristics

**Figure 2:**
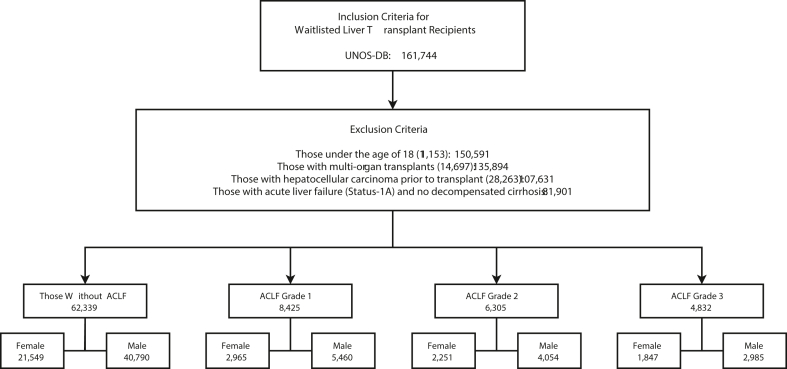
Inclusion and exclusion criteria and final study population.

This study included 84,279 waitlisted patients, comprising 62,339 patients without ACLF, 8425 with ACLF grade 1, 6305 with ACLF grade 2, and 7210 with ACLF grade 3. Figure 2 details the patient selection process.

Table 1, Table 2, Table 3, Table 4 illustrate baseline characteristics that were compared between females and males within each ACLF grade using SMDs, with SMD >0.10 indicating meaningful imbalance. Overall, demographic variables were well balanced by sex across ACLF strata. The results will be represented as the following: (male vs female). Mean age was similar between sexes in the no-ACLF group (55.00 ± 8.39 vs 55.30 ± 9.48 years), ACLF grade 1 (54.40 ± 9.15 vs 54.30 ± 10.40 years), ACLF grade 2 (51.80 ± 9.78 vs 52.10 ± 10.80 years), and ACLF grade 3 (51.70 ± 9.93 vs 51.60 ± 11.10 years), with corresponding SMDs below thresholds for meaningful imbalance. Body mass index (BMI) was likewise similar between sexes across ACLF grades (no ACLF: 29.30 ± 5.34 vs 29.00 ± 6.31 kg/m^2^; ACLF grade 1: 29.30 ± 5.75 vs 29.30 ± 6.68 kg/m^2^; ACLF grade 2: 29.70 ± 6.07 vs 30.20 ± 7.16 kg/m^2^; ACLF grade 3: 30.20 ± 6.25 vs 30.70 ± 7.53 kg/m^2^ for males vs females), without meaningful imbalance.

In contrast, liver disease etiologies demonstrated a consistent and clinically meaningful sex-based imbalance across ACLF grades. Alcohol-associated liver disease was more prevalent among males across all strata, including no ACLF (42.08% vs 24.94%), ACLF grade 1 (50.48% vs 32.48%), ACLF grade 2 (57.94% vs 39.05%), and ACLF grade 3 (55.91% vs 37.25%). Similarly, hepatitis C was more common among males (no ACLF: 45.51% vs 35.44%; ACLF grade 1: 34.89% vs 24.01%; ACLF grade 2: 30.04% vs 21.99%; ACLF grade 3: 34.17% vs 24.74%), as was hepatitis B (no ACLF: 3.11% vs 1.68%; ACLF grade 1: 3.74% vs 1.45%; ACLF grade 2: 5.11% vs 2.80%; ACLF grade 3: 7.67% vs 2.65%). These differences exceeded SMD thresholds for meaningful imbalance across most ACLF grades. In contrast, diabetes prevalence was similar by sex across strata (no ACLF: 23.00% vs 24.59%; ACLF grade 1: 22.47% vs 22.39%; ACLF grade 2: 17.56% vs 19.64%; ACLF grade 3: 18.89% vs 16.84% for females vs males), without clinically meaningful imbalance.

Measures of liver disease severity demonstrated sex-based differences of clinical relevance. Model for End-Stage Liver Disease (MELD) scores were higher among males in the no-ACLF group (15.20 ± 4.58 vs 14.90 ± 4.74), ACLF grade 2 (33.00 ± 6.07 vs 32.20 ± 5.76), and ACLF grade 3 (37.90 ± 7.63 vs 35.40 ± 7.49), while values were similar in ACLF grade 1 (26.00 ± 5.23 vs 26.10 ± 5.12). These differences were largely driven by renal dysfunction, as serum creatinine was consistently higher among males across all ACLF strata (no ACLF: 1.03 ± 0.31 vs 0.93 ± 0.32 mg/dL; ACLF grade 1: 1.96 ± 1.30 vs 1.77 ± 1.17 mg/dL; ACLF grade 2: 2.19 ± 1.54 vs 1.89 ± 1.33 mg/dL; ACLF grade 3: 2.79 ± 1.67 vs 2.18 ± 1.38 mg/dL), representing clinically meaningful imbalance. International normalized ratio values were similar between sexes across ACLF grades and did not demonstrate meaningful imbalance (no ACLF: 1.48 ± 0.37 vs 1.48 ± 0.36; ACLF grade 1: 2.09 ± 0.81 vs 2.14 ± 2.02; ACLF grade 2: 2.70 ± 1.13 vs 2.78 ± 1.13; ACLF grade 3: 3.07 ± 1.48 vs 3.08 ± 1.66 for males vs females).

Portal hypertension–related features differed by sex. Moderate ascites was more common among males across all ACLF grades (no ACLF: 22.10% vs 19.02%; ACLF grade 1: 45.92% vs 41.15%; ACLF grade 2: 51.33% vs 46.47%; ACLF grade 3: 63.28% vs 59.45%), meeting thresholds for meaningful imbalance. Hepatic encephalopathy severity varied by ACLF grade, with less consistent sex-based patterns; severe encephalopathy (grades 3–4) occurred at similar frequencies in no ACLF (35.33% vs 36.79%), ACLF grade 2 (19.49% vs 20.21%), and ACLF grade 3 (9.65% vs 7.69%), with only select strata demonstrating meaningful imbalance.

Laboratory markers of cholestasis showed ACLF-dependent sex differences. Total bilirubin levels were higher among females at lower disease severity (no ACLF: 3.44 ± 3.57 vs 3.25 ± 3.14 mg/dL; ACLF grade 1: 10.70 ± 9.83 vs 9.36 ± 9.53 mg/dL) but higher among males at advanced ACLF grades (ACLF grade 2: 19.20 ± 12.80 vs 18.30 ± 11.80 mg/dL; ACLF grade 3: 24.50 ± 13.20 vs 22.50 ± 12.70 mg/dL), with meaningful imbalance observed in advanced disease.

Finally, indicators of critical illness differed by sex at higher ACLF grades. Mechanical ventilation was uncommon at waitlisting in no ACLF and ACLF grade 1, remained low in ACLF grade 2 (2.24% vs 2.44% for males vs females) but was frequent in ACLF grade 3, where females had a higher ventilator rate than males (41.96% vs 31.86%), representing a clinically meaningful imbalance.

### Clinical Outcomes

Table 5 provides competing-risk regression results for primary waitlist outcomes (death vs LT) across ACLF grades. In the fully adjusted model (model 4), male candidates were associated with lower all-cause waitlist mortality compared to female candidates without ACLF (subdistribution hazard ratio [SHR]: 0.87; 95% confidence interval [CI]: 0.83–0.90; *P* < .001), ACLF grade 1 (SHR: 0.83; 95% CI: 0.73–0.95; *P* = .006), and ACLF grade 2 (SHR: 0.81; 95% CI: 0.70–0.95; *P* = .008). No statistically significant sex difference in waitlist mortality was observed in ACLF grade 3 (SHR: 0.92; 95% CI: 0.80–1.06; *P* = .23).

**Table 5:** Competing Risk Regression Analysis of Sex and Waitlist Outcomes (Death vs Liver Transplantation) Across Acute-on-Chronic Liver Failure Grades

Without acute-on-chronic liver failure				Acute-on-chronic liver failure grade 1				Acute-on-chronic liver failure grade 2				Acute-on-chronic liver failure grade 3			
(A) All-cause mortality				(A) All-cause mortality				(A) All-cause mortality				(A) All-cause mortality			
Model	*P* value	SHR	95% CI	Model	*P* value	SHR	95% CI	Model	*P* value	SHR	95% CI	Model	*P* value	SHR	95% CI
1	<.001^c^	0.88	(0.84–0.92)	1	.002^b^	.82	(0.72–0.93)	1	.04^a^	0.86	(0.74–0.99)	1	.17	0.91	(0.80–1.04)
2	<.001^c^	0.87	(0.83–0.91)	2	.007^b^	.83	(0.73–0.95)	2	.008^b^	0.81	(0.69–0.95)	2	.35	0.94	(0.81–1.08)
3	<.001^c^	0.87	(0.83–0.90)	3	.006^b^	.83	(0.73–0.95)	3	.008^b^	0.81	(0.70–0.95)	3	.23	0.92	(0.80–1.06)

(B) Transplantation				(B) Transplantation				(B) Transplantation				(B) Transplantation			
Model	*P* value	SHR	95% CI	Model	*P* value	SHR	95% CI	Model	*P* value	SHR	95% CI	Model	*P* value	SHR	95% CI
1	<.001^c^	1.17	(1.15–1.20)	1	<.001^c^	1.11	(1.05–1.17)	1	<.001^c^	1.11	(1.05–1.18)	1	<.001^c^	1.16	(1.08–1.24)
2	<.001^c^	1.23	(1.20–1.26)	2	.003^b^	1.09	(1.03–1.15)	2	<.001^c^	1.13	(1.06–1.20)	2	<.001^c^	1.14	(1.06–1.23)
3	<.001^c^	1.21	(1.18–1.24)	3	<.001^c^	1.11	(1.05–1.18)	3	.001^b^	1.11	(1.04–1.18)	3	.004^b^	1.12	(1.04–1.21)

FM, final model.

Model 1 includes VOI (variable of interest) and demographics; model 2 includes model 1 terms with the addition of comorbidities and liver disease etiologies; model 3 includes model 2 terms with the addition of hepatic variables, MELD score, and liver laboratory markers; model 4 includes model 3 terms with the addition of donor demographics.

a *P* < .05.

b *P* < .01.

c *P* < .001.

For LT, male sex was associated with higher transplant rates across all strata in model 4: without ACLF (SHR: 1.21; 95% CI: 1.18–1.24; *P* < .001), ACLF grade 1 (SHR: 1.11; 95% CI: 1.05–1.18; *P* < .001), ACLF grade 2 (SHR: 1.11; 95% CI: 1.04–1.18; *P* = .001), and ACLF grade 3 (SHR: 1.12; 95% CI: 1.04–1.21; *P* = .003).

## Discussion

This study explores the sex disparities that exist in LT waitlist outcomes among patients with ACLF. Prior research has consistently highlighted that females were generally less likely to receive an LT and face a higher risk of mortality while on the waitlist compared to males.^19^^,^^24^^,^^25^ Females were also more frequently delisted from the LT waitlist due to being classified as “too sick” for transplantation.^26^ Additionally, Sundaram et al found that females had significantly higher 28-day and 90-day mortality on the LT waitlist for ACLF compared to males.^27^ However, there are a limited number of studies to date that have explored these sex disparities in relation to ACLF grade. Our findings using competing risk regression show that females had lower transplant rates across all ACLF grades. Females also demonstrated a significantly higher waitlist mortality in ACLF grades 1 and 2.

Notably, although both cause-specific and competing-risk regression models consistently showed lower transplantation rates among women across ACLF grades, sex-based differences in waitlist mortality were observed only in competing-risk analyses. This divergence suggests that the excess cumulative mortality observed among women—particularly in ACLF grades 0–2—may reflect prolonged waitlist exposure due to reduced access to transplantation rather than an increased instantaneous risk of death.

In ACLF grades 1 and 2, where the organ failure burden is lower, the underestimation of disease severity in women by MELD-sodium (Na) may significantly delay prioritization for transplant. The higher mortality and lower LT rates in females compared to males across these grades likely reflects the inherent limitations of the MELD-based allocation system. Studies have shown that women, due to lower muscle mass, tend to have lower serum creatinine levels, which results in an underestimation of renal dysfunction. As a result, women often receive lower MELD scores compared to men with similar degrees of renal impairment, delaying their prioritization for LT.^19^^,^^28^^,^^29^ Additionally, men generally have lower serum sodium levels, which may further exacerbate sex disparities when the MELD-Na score is used.^19^ Body size also plays an important role in organ allocation. Women tend to have smaller statures and abdominal cavities, which can result in donor-recipient size mismatches.^30^^,^^31^ Consequently, larger livers are more likely to be allocated to men. Previous studies have demonstrated that shorter individuals, particularly those under 175 cm, are 10%–15% less likely to receive liver transplants compared to taller individuals.^19^^,^^32^^,^^33^ To be consistent with this literature, sensitivity analyses substituting height and weight for BMI were performed and demonstrated no statistically significant sex-based difference in transplant rate after adjustment for height and weight, supporting body size may be a key mediator of observed sex differences. These factors, taken together, place women at a considerable disadvantage on the transplant waitlist, especially in ACLF grades 1 and 2, where lower MELD-Na scores are more common despite high short-term mortality risk. Therefore, the implementation of MELD 3.0 by UNOS in July 2023 is a significant step toward addressing sex-related disparities, as it includes female sex as a variable.^29^ However, it remains to be seen to what extent these disparities will be minimized by this change.

Research indicates a higher prevalence of sarcopenia and frailty in females compared to males, which contributes to poorer waitlist outcomes observed.^34^^,^^35^ Additionally, Sundaram et al identified metabolic dysfunction–associated steatotic liver disease as the most common underlying liver disease in females presenting with ACLF, an etiology linked to higher waitlist mortality rates compared to other liver diseases.^27^ Female sex has also been associated with a higher likelihood of being delisted from the liver transplant waitlist.^26^ However, it is important to note that delisting in the context of ACLF has not been specifically studied, and our findings did not directly implicate sarcopenia or metabolic dysfunction–associated steatotic liver disease as contributing factors. Despite this, these remain plausible explanations for the observed disparities. Interestingly, in ACLF grade 3, where the severity of the condition is at its peak, sex differences in waitlist outcomes became less pronounced. Previous studies have reported that the 3-month mortality rate for patients with ACLF grade 3 approaches 80% without LT.^36^ The overwhelming burden of multi-organ failure in this advanced grade likely becomes the primary determinant of mortality, potentially diminishing the influence of sex-specific factors.^2^^,^^9^

Our study is subject to the inherent limitations of retrospective cohort analyses, including selection bias. ACLF is a dynamic condition with the potential for fluctuations over time.^9^^,^^14^ While ACLF grade information was available at the time of listing and transplant, the data did not track how the condition evolved between these points. Candidate body size can be characterized using several approaches. In the primary analyses, BMI was used, with sensitivity analyses incorporating height and weight separately; however, more granular measures of size compatibility, such as donor imaging or graft volume estimates, were not available in this dataset. Another challenge lies in the potential misclassification of ACLF grade within the registry data as highlighted by Lee et al^37^ UNOS-derived ACLF definitions demonstrate only ∼70% concordance with chart-review–based ACLF classification, the current gold standard. Their study underscores that UNOS does not consistently capture ACLF accurately, leading to misclassification of organ failures—particularly circulatory and respiratory failure. For example, circulatory and respiratory failure are identified based on surrogate markers such as vasopressor use and mechanical ventilation, which may not always accurately reflect the underlying condition. These considerations support a cautious interpretation of registry-based ACLF measures.

## Conclusion

Competing risk analysis demonstrated higher waitlist mortality among women than men in ACLF grades 1 and 2, potentially reflecting delayed transplant prioritization among female candidates.

## Supplementary Materials

Supplementary Tables 1–3

Extended PDF
