# Supplementary material for: The Clinical Implications of Sex on Waitlist Outcomes in Patients With Acute-on-Chronic Liver Failure
Source: Gastro Hep Adv. 2026 Apr 13;5(7):100970. doi: 10.1016/j.gastha.2026.100970 (PMC13207543; doi:10.1016/j.gastha.2026.100970)
Supplement: Supplementary Figure 1 [file mmc1.pdf]

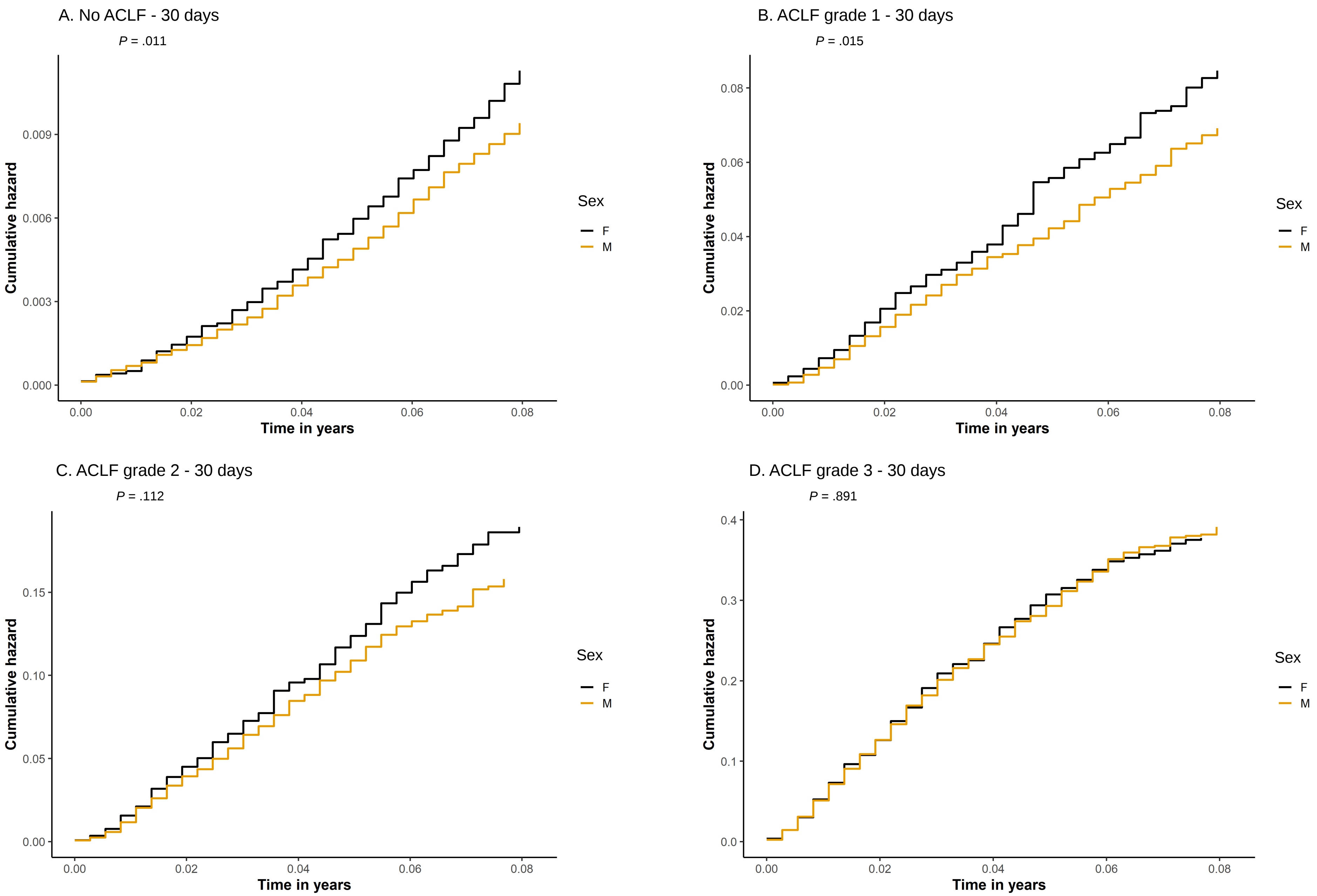

Supplementary figure 1 (A) through (D) depict the 90 day cumulative hazard for all-cause mortality among patients on the liver transplant waitlist without acute-on-chronic liver failure (ACLF) and with ACLF grades 1–3, stratified by sex.
