# Supplementary material for: The Clinical Implications of Sex on Waitlist Outcomes in Patients With Acute-on-Chronic Liver Failure
Source: Gastro Hep Adv. 2026 Apr 13;5(7):100970. doi: 10.1016/j.gastha.2026.100970 (PMC13207543; doi:10.1016/j.gastha.2026.100970)

Multivariable Forest Plot using Sex as Exposure and Post-Waitlist Mortality as Outcome in ACLF Grade 0

Variables	OR	95% CI	*P* value
Sex: Male (vs. Female)	0.94	0.90 - 0.98	.009
Race: Black (vs. White)	0.98	0.89 - 1.08	.674
Race: Hispanic (vs. White)	1.13	1.07 - 1.19	< .001
Race: Asian and Pacific Islander (vs. White)	0.95	0.82 - 1.10	.470
Race: Other (vs. White)	0.93	0.77 - 1.12	.442
Age	1.02	1.02 - 1.02	< .001
BMI	1.00	1.00 - 1.01	.063
Hepatitis B	0.85	0.72 - 1.00	.050
Hepatitis C	1.30	1.23 - 1.37	< .001
Autoimmune Hepatitis	1.16	1.05 - 1.29	.005
Alcoholic Associated Liver Disease	1.01	0.95 - 1.06	.819
MASH	1.19	1.11 - 1.28	< .001
Diabetes	1.11	1.06 - 1.17	< .001
MELD Score	1.08	1.08 - 1.09	< .001
TIPS	0.93	0.87 - 1.00	.047

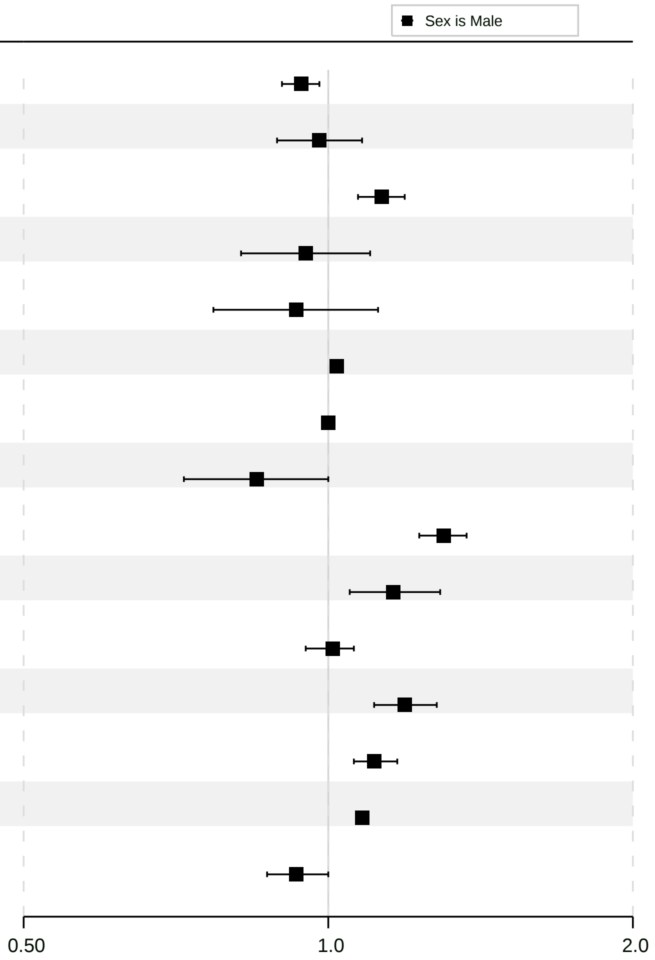

Supplement: Supplementary Figure 3 [file mmc3.pdf]
