# Supplementary material for: The Clinical Implications of Sex on Waitlist Outcomes in Patients With Acute-on-Chronic Liver Failure
Source: Gastro Hep Adv. 2026 Apr 13;5(7):100970. doi: 10.1016/j.gastha.2026.100970 (PMC13207543; doi:10.1016/j.gastha.2026.100970)

Multivariable Forest Plot using Sex as Exposure and Post-Waitlist Mortality as Outcome in ACLF Grade 1

Variables	OR	95% CI	*P* value
Sex: Male (vs. Female)	0.88	0.77 - 1.00	.053
Race: Black (vs. White)	0.94	0.74 - 1.20	.622
Race: Hispanic (vs. White)	0.96	0.81 - 1.15	.676
Race: Asian and Pacific Islander (vs. White)	0.83	0.54 - 1.29	.416
Race: Other (vs. White)	1.30	0.82 - 2.08	.268
Age	1.02	1.01 - 1.03	< .001
BMI	1.00	0.99 - 1.01	.885
Hepatitis B	1.20	0.81 - 1.77	.360
Hepatitis C	1.06	0.90 - 1.24	.480
Autoimmune Hepatitis	1.21	0.93 - 1.59	.153
Alcoholic Associated Liver Disease	0.85	0.72 - 0.99	.038
MASH	0.83	0.67 - 1.02	.080
Diabetes	1.07	0.92 - 1.24	.382
MELD Score	1.05	1.03 - 1.06	< .001
TIPS	0.93	0.74 - 1.16	.496

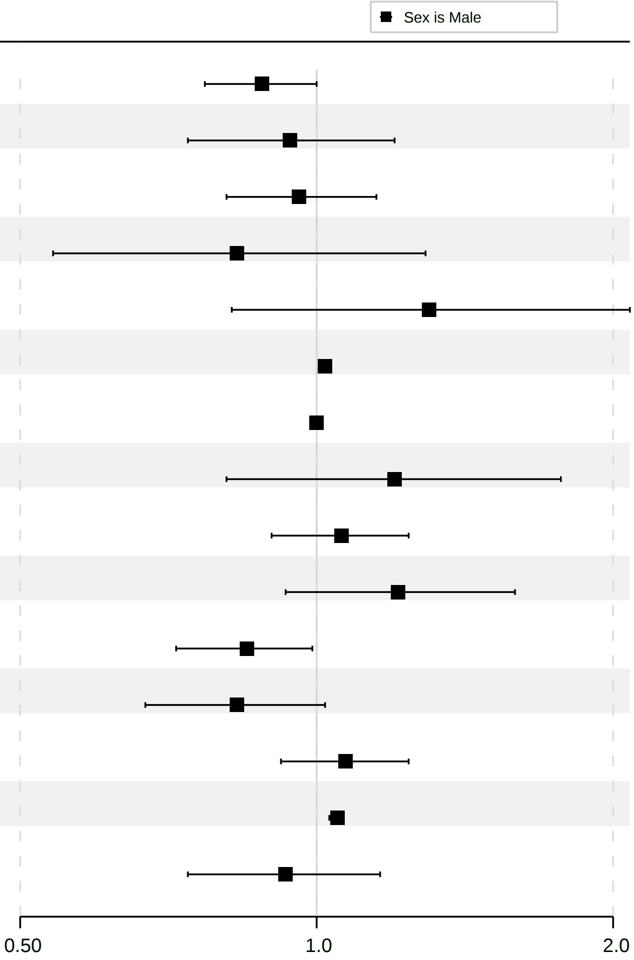

Supplement: Supplementary Figure 4 [file mmc4.pdf]
