# Supplementary material for: The Clinical Implications of Sex on Waitlist Outcomes in Patients With Acute-on-Chronic Liver Failure
Source: Gastro Hep Adv. 2026 Apr 13;5(7):100970. doi: 10.1016/j.gastha.2026.100970 (PMC13207543; doi:10.1016/j.gastha.2026.100970)

Multivariable Forest Plot using Sex as Exposure and Post-Waitlist Mortality as Outcome in ACLF Grade 2

Variables	OR	95% CI	*P* value
Sex: Male (vs. Female)	0.87	0.74 - 1.01	.072
Race: Black (vs. White)	1.30	1.03 - 1.64	.029
Race: Hispanic (vs. White)	1.01	0.82 - 1.23	.945
Race: Asian and Pacific Islander (vs. White)	0.98	0.64 - 1.51	.928
Race: Other (vs. White)	0.82	0.45 - 1.49	.513
Age	1.02	1.01 - 1.03	< .001
BMI	1.01	1.00 - 1.02	.071
Hepatitis B	1.29	0.86 - 1.92	.218
Hepatitis C	1.14	0.94 - 1.37	.179
Autoimmune Hepatitis	0.97	0.69 - 1.38	.878
Alcoholic Associated Liver Disease	1.04	0.86 - 1.25	.714
MASH	0.89	0.67 - 1.17	.390
Diabetes	1.00	0.82 - 1.21	.983
MELD Score	1.03	1.02 - 1.05	< .001
TIPS	0.71	0.52 - 0.97	.033

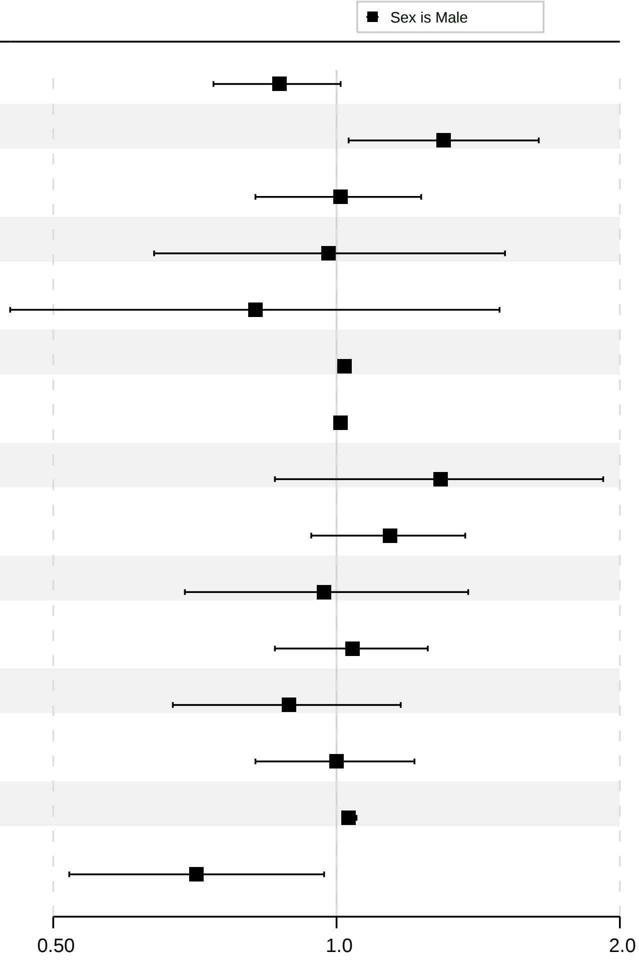

Supplement: Supplementary Figure 5 [file mmc5.pdf]
