# Supplementary material for: The Clinical Implications of Sex on Waitlist Outcomes in Patients With Acute-on-Chronic Liver Failure
Source: Gastro Hep Adv. 2026 Apr 13;5(7):100970. doi: 10.1016/j.gastha.2026.100970 (PMC13207543; doi:10.1016/j.gastha.2026.100970)

Multivariable Forest Plot using Sex as Exposure and Post-Waitlist Mortality as Outcome in ACLF Grade 3

Variables	OR	95% CI	*P* value
Sex: Male (vs. Female)	0.98	0.85 - 1.13	.780
Race: Black (vs. White)	0.72	0.57 - 0.91	.005
Race: Hispanic (vs. White)	0.86	0.72 - 1.03	.111
Race: Asian and Pacific Islander (vs. White)	0.88	0.63 - 1.24	.470
Race: Other (vs. White)	0.57	0.33 - 1.00	.048
Age	1.00	1.00 - 1.01	.158
BMI	1.00	0.99 - 1.01	.839
Hepatitis B	1.16	0.84 - 1.59	.363
Hepatitis C	1.05	0.89 - 1.24	.568
Autoimmune Hepatitis	1.14	0.87 - 1.51	.349
Alcoholic Associated Liver Disease	0.81	0.69 - 0.96	.015
MASH	0.90	0.70 - 1.15	.409
Diabetes	0.89	0.74 - 1.06	.196
MELD Score	1.02	1.02 - 1.03	< .001
TIPS	1.02	0.79 - 1.30	.900

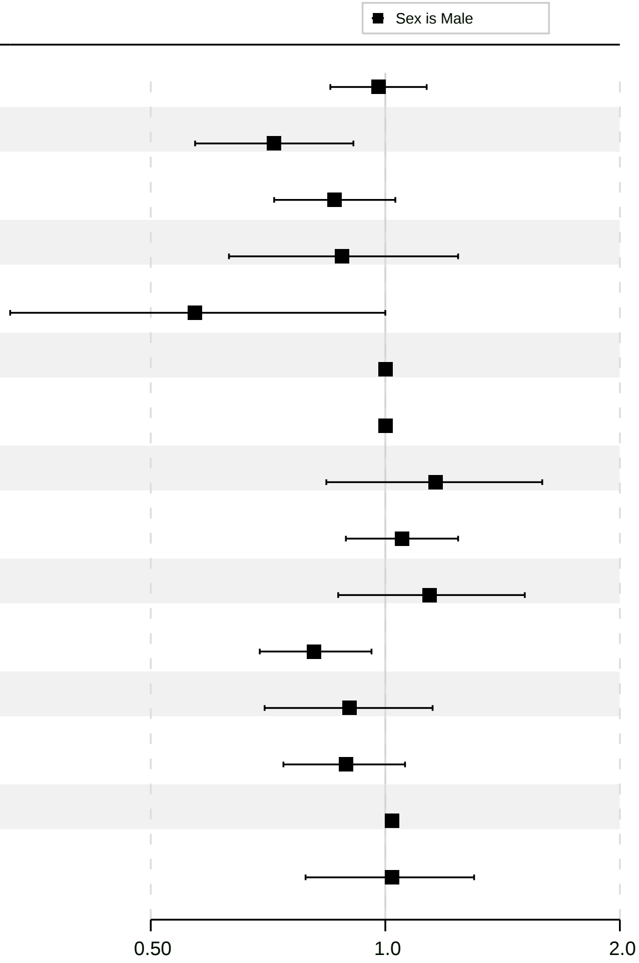

Supplement: Supplementary Figure 6 [file mmc6.pdf]
