# Supplementary figures and images for: The Clinical Implications of Sex on Waitlist Outcomes in Patients With Acute-on-Chronic Liver Failure
Source: Gastro Hep Adv. 2026 Apr 13;5(7):100970. doi: 10.1016/j.gastha.2026.100970 (PMC13207543; doi:10.1016/j.gastha.2026.100970)

Distribution of Missing Rates Among the Variables

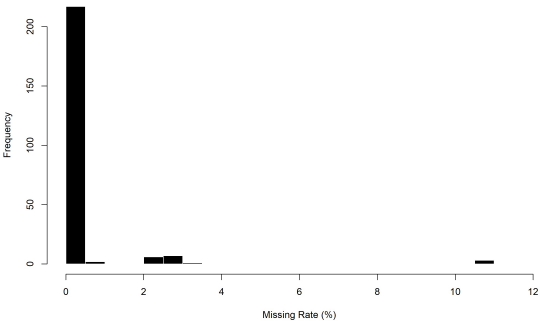

Supplement: Supplementary Figure 7 [file mmc7.pdf]
