# Supplementary material for: The Clinical Implications of Sex on Waitlist Outcomes in Patients With Acute-on-Chronic Liver Failure
Source: Gastro Hep Adv. 2026 Apr 13;5(7):100970. doi: 10.1016/j.gastha.2026.100970 (PMC13207543; doi:10.1016/j.gastha.2026.100970)
Supplement: Supplementary Tables 1–3 [file mmc8.pdf]

Supplementary Table 1: Sequential Cox Regression Analysis in Waitlisted Patients Using Sex as a Prognostic Risk Factor for All-Cause Mortality and Transplantation: Varying Severities of Acute-on-Chronic Liver Failure

Without Acute-on-Chronic Liver Failure

(A) All-Cause Mortality

	Raw Incidence	Incidence Rates per 1000 Person-Years	
Male	5513	40.73	(39.68 - 41.80)
Female	3295	42.35	(40.95 - 43.79)

Sequential Cox Regression Analysis

Model	*P* value	aHR	95% CI
1	.03 *	0.95	(0.91 - 1.00)
2	.08	0.96	(0.92 - 1.00)
3	.008 **	0.94	(0.90 - 0.98)

(B) Transplantation

	Raw Incidence	Incidence Rates per 1000 Person-Years	
Male	21259	157.06	(155.12 - 159.01)
Female	10025	128.85	(126.50 - 131.22)

Sequential Cox Regression Analysis

Model	*P* value	aHR	95% CI
1	< .001 ***	1.17	(1.14 - 1.20)
2	< .001 ***	1.23	(1.20 - 1.26)
3	< .001 ***	1.20	(1.17 - 1.23)

Acute-on-Chronic Liver Failure Grade 1

(A) All-Cause Mortality

	Raw Incidence	Incidence Rates per 1000 Person-Years	
Male	616	80.29	(74.30 - 86.59)
Female	403	97.88	(88.97 - 107.37)

Sequential Cox Regression Analysis

Model	*P* value	aHR	95% CI
1	.02 *	0.86	(0.75 - 0.97)
2	.03 *	0.87	(0.76 - 0.99)
3	.05	0.88	(0.77 - 1.00)

(B) Transplantation

	Raw Incidence	Incidence Rates per 1000 Person-Years	
Male	3856	502.58	(491.33 - 513.83)
Female	1993	484.08	(468.71 - 499.47)

Sequential Cox Regression Analysis

Model	*P* value	aHR	95% CI
1	.004 **	1.08	(1.03 - 1.14)
2	.03 *	1.07	(1.01 - 1.13)
3	.002 **	1.09	(1.03 - 1.16)

Acute-on-Chronic Liver Failure Grade 2

(A) All-Cause Mortality

	Raw Incidence	Incidence Rates per 1000 Person-Years	
Male	439	122.08	(111.55 - 133.23)
Female	290	122.68	(109.71 - 136.58)

Sequential Cox Regression Analysis

Model	*P* value	aHR	95% CI
1	.24	0.92	(0.79 - 1.06)
2	.11	0.88	(0.75 - 1.03)
3	.08	0.87	(0.74 - 1.02)

(B) Transplantation

	Raw Incidence	Incidence Rates per 1000 Person-Years	
Male	3096	860.96	(849.22 - 872.11)
Female	1614	682.76	(663.57 - 701.49)

Sequential Cox Regression Analysis

Model	*P* value	aHR	95% CI
1	.001 **	1.11	(1.04 - 1.17)
2	< .001 ***	1.12	(1.05 - 1.19)
3	.004 **	1.10	(1.03 - 1.17)

Acute-on-Chronic Liver Failure Grade 3

(A) All-Cause Mortality

	Raw Incidence	Incidence Rates per 1000 Person-Years	
Male	540	180.63	(166.99 - 194.90)
Female	362	156.56	(141.98 - 172.02)

Sequential Cox Regression Analysis

Model	*P* value	aHR	95% CI
1	.93	1.01	(0.88 - 1.15)
2	.67	1.03	(0.90 - 1.19)
3	.78	0.98	(0.85 - 1.13)

(B) Transplantation

	Raw Incidence	Incidence Rates per 1000 Person-Years	
Male	2017	674.70	(657.57 - 691.48)
Female	1135	490.88	(470.31 - 511.48)

Sequential Cox Regression Analysis

Model	*P* value	aHR	95% CI
1	< .001 ***	1.18	(1.09 - 1.27)
2	< .001 ***	1.16	(1.08 - 1.26)
3	.003 **	1.12	(1.04 - 1.21)

* p < 0.05, ** p < 0.01, *** p < 0.001

† FM indicates Final Model

Footnote: *Model 1 includes VOI (variable of interest) and demographics; Model 2 includes Model 1 terms with the addition of comorbidities, and liver disease etiologies; Model 3 includes Model 2 terms with the addition of hepatic variables, MELD score, and liver laboratory markers; Model 4 includes Model 3 terms with the addition of donor demographics

Supplementary Table 2. Sequential Cox Regression Analysis in Waitlisted Patients Using Sex as a Prognostic Risk Factor for Specific Causes of Death: Various Grades of Acute-on-Chronic Liver Failure

Without Acute-on-Chronic Liver Failure				Acute-on-Chronic Liver Failure Grade 1				Acute-on-Chronic Liver Failure Grade 2				Acute-on-Chronic Liver Failure Grade 3			
**(A) Death due to General Cardiac Causes**				**(A) Death due to General Cardiac Causes**				**(A) Death due to General Cardiac Causes**				**(A) Death due to General Cardiac Causes**			
	**Raw Incidence**	**Incidence Rates per 1000 Person-Years**			**Raw Incidence**	**Incidence Rates per 1000 Person-Years**			**Raw Incidence**	**Incidence Rates per 1000 Person-Years**			**Raw Incidence**	**Incidence Rates per 1000 Person-Years**	
Male	407	3.01	(2.72 - 3.31)	Male	47	6.13	(4.50 - 8.14)	Male	38	10.57	(7.49 - 14.48)	Male	48	16.06	(11.86 - 21.23)
Female	240	3.08	(2.71 - 3.50)	Female	30	7.29	(4.92 - 10.39)	Female	27	11.42	(7.54 - 16.58)	Female	24	10.38	(6.66 - 15.41)
**Sequential Cox Regression Analyis**				**Sequential Cox Regression Analysis**				**Sequential Cox Regression Analysis**				**Sequential Cox Regression Analysis**			
**Model**	***P* value**	**aHR**	**95% CI**	**Model**	***P* value**	**aHR**	**95% CI**	**Model**	***P* value**	**aHR**	**95% CI**	**Model**	***P* value**	**aHR**	**95% CI**
1	.74	0.97	(0.83 - 1.14)	1	.59	0.88	(0.56 - 1.40)	1	.58	0.87	(0.53 - 1.43)	1	.24	1.34	(0.82 - 2.20)
2	.87	0.99	(0.83 - 1.16)	2	.73	0.92	(0.57 - 1.48)	2	.52	0.84	(0.50 - 1.42)	2	.43	1.23	(0.74 - 2.06)
3	.66	0.96	(0.82 - 1.14)	3	.77	0.93	(0.58 - 1.50)	3	.44	0.81	(0.48 - 1.38)	3	.50	1.19	(0.71 - 2.01)
**(B) Death due to Cardiac Arrest**				**(B) Death due to Cardiac Arrest**				**(B) Death due to Cardiac Arrest**				**(B) Death due to Cardiac Arrest**			
	**Raw Incidence**	**Incidence Rates per 1000 Person-Years**		**Exposure Variable**	**Raw Incidence**	**Incidence Rates per 1000 Person-Years**		**Exposure Variable**	**Raw Incidence**	**Incidence Rates per 1000 Person-Years**		**Exposure Variable**	**Raw Incidence**	**Incidence Rates per 1000 Person-Years**	
Male	292	2.16	(1.92 - 2.42)	Male	32	4.17	(2.85 - 5.88)	Male	25	6.95	(4.50 - 10.25)	Male	36	12.04	(8.45 - 16.63)
Female	165	2.12	(1.81 - 2.47)	Female	26	6.32	(4.13 - 9.24)	Female	23	9.73	(6.18 - 14.56)	Female	18	7.78	(4.62 - 12.28)
**Sequential Cox Regression Analysis**				**Sequential Cox Regression Analysis**				**Sequential Cox Regression Analysis**				**Sequential Cox Regression Analysis**			
**Model**	***P value***	**aHR**	**95% CI**	**Model**	***P* value**	**aHR**	**95% CI**	**Model**	***P* value**	**aHR**	**95% CI**	**Model**	***P* value**	**aHR**	**95% CI**
1	.86	1.02	(0.84 - 1.23)	1	.18	0.70	(0.42 - 1.17)	1	.15	0.66	(0.37 - 1.16)	1	.29	1.36	(0.77 - 2.40)
2	.63	1.05	(0.86 - 1.28)	2	.24	0.72	(0.42 - 1.24)	2	.10	0.60	(0.33 - 1.10)	2	.41	1.29	(0.71 - 2.33)
3	.79	1.03	(0.84 - 1.25)	3	.27	0.74	(0.43 - 1.26)	3	.08	0.58	(0.32 - 1.06)	3	.45	1.26	(0.69 - 2.30)
**(C) Death due to Myocardial Infarction**				**(C) Death due to Myocardial Infarction**				**(C) Death due to Myocardial Infarction**				**(C) Death due to Myocardial Infarction**			
	**Raw Incidence**	**Incidence Rates per 1000 Person-Years**			**Raw Incidence**	**Incidence Rates per 1000 Person-Years**			**Raw Incidence**	**Incidence Rates per 1000 Person-Years**			**Raw Incidence**	**Incidence Rates per 1000 Person-Years**	
Male	54	0.40	(0.30 - 0.52)	Male	5	0.65	(0.21 - 1.52)	Male	3	0.83	(0.17 - 2.44)	Male	2	0.67	(0.08 - 2.41)
Female	27	0.35	(0.23 - 0.50)	Female	1	0.24	(0.01 - 1.35)	Female	1	0.42	(0.01 - 2.35)	Female	1	0.43	(0.01 - 2.41)
**Sequential Cox Regression Analysis**				**Sequential Cox Regression Analysis**				**Sequential Cox Regression Analysis**				**Sequential Cox Regression Analysis**			
**Model**	***P* value**	**aHR**	**95% CI**	**Model**	***P* value**	**aHR**	**95% CI**	**Model**	***P* value**	**aHR**	**95% CI**	**Model**	***P* value**	**aHR**	**95% CI**
1	.53	1.16	(0.73 - 1.84)	1	.33	2.95	(0.34 - 25.55)	1	.60	1.84	(0.19 - 17.84)	1	.82	1.33	(0.12 - 14.70)
2	.66	1.11	(0.69 - 1.80)	2	.33	3.00	(0.33 - 27.18)	2	.64	1.72	(0.17 - 17.05)	2	.79	1.39	(0.12 - 16.16)
3	.72	1.09	(0.67 - 1.76)	3	.33	2.99	(0.33 - 27.09)	3	.76	1.43	(0.14 - 15.00)	3	.87	1.23	(0.10 - 14.71)
**(D) Death due to Gastrointestinal Hemorrhage**				**(D) Death due to Gastrointestinal Hemorrhage**				**(D) Death due to Gastrointestinal Hemorrhage**				**(D) Death due to Gastrointestinal Hemorrhage**			
	**Raw Incidence**	**Incidence Rates per 1000 Person-Years**		**Exposure Variable**	**Raw Incidence**	**Incidence Rates per 1000 Person-Years**		**Exposure Variable**	**Raw Incidence**	**Incidence Rates per 1000 Person-Years**		**Exposure Variable**	**Raw Incidence**	**Incidence Rates per 1000 Person-Years**	
Male	31	0.23	(0.16 - 0.33)	Male	2	0.26	(0.03 - 0.94)	Male	5	1.39	(0.45 - 3.24)	Male	0	0.00	(0.00 - 1.23)
Female	19	0.24	(0.15 - 0.38)	Female	3	0.73	(0.15 - 2.13)	Female	3	1.27	(0.26 - 3.70)	Female	5	2.16	(0.70 - 5.04)
**Sequential Cox Regression Analysis**				**Sequential Cox Regression Analysis**				**Sequential Cox Regression Analysis**				**Sequential Cox Regression Analysis**			
**Model**	***P* value**	**aHR**	**95% CI**	**Model**	***P* value**	**aHR**	**95% CI**	**Model**	***P* value**	**aHR**	**95% CI**	**Model**	***P* value**	**aHR**	**95% CI**
1	.95	0.98	(0.55 - 1.74)	1	.29	0.38	(0.06 - 2.29)	1	.91	0.92	(0.22 - 3.85)	1	1.00	0.00	(0.00 - Inf)
2	.92	1.03	(0.57 - 1.87)	2	.35	0.41	(0.06 - 2.63)	2	.65	0.71	(0.17 - 3.02)	2	1.00	0.00	(0.00 - Inf)
3	.96	1.02	(0.56 - 1.84)	3	.37	0.43	(0.07 - 2.76)	3	.51	0.62	(0.15 - 2.58)	3	1.00	0.00	(0.00 - Inf)
**(E) Death due to General Hemorrhagic Causes**				**(E) Death due to General Hemorrhagic Causes**				**(E) Death due to General Hemorrhagic Causes**				**(E) Death due to General Hemorrhagic Causes**			
	**Raw Incidence**	**Incidence Rates per 1000 Person-Years**			**Raw Incidence**	**Incidence Rates per 1000 Person-Years**			**Raw Incidence**	**Incidence Rates per 1000 Person-Years**			**Raw Incidence**	**Incidence Rates per 1000 Person-Years**	
Male	422	3.12	(2.83 - 3.43)	Male	56	7.30	(5.52 - 9.47)	Male	43	11.96	(8.67 - 16.07)	Male	45	15.05	(11.00 - 20.09)
Female	232	2.98	(2.61 - 3.39)	Female	36	8.74	(6.13 - 12.09)	Female	37	15.65	(11.04 - 21.51)	Female	34	14.71	(10.20 - 20.49)
**Sequential Cox Regression Analysis**				**Sequential Cox Regression Analysis**				**Sequential Cox Regression Analysis**				**Sequential Cox Regression Analysis**			
**Model**	***P* value**	**aHR**	**95% CI**	**Model**	***P* value**	**aHR**	**95% CI**	**Model**	***P* value**	**aHR**	**95% CI**	**Model**	***P* value**	**aHR**	**95% CI**
1	.56	1.05	(0.89 - 1.23)	1	.53	0.87	(0.57 - 1.33)	1	.09	0.68	(0.44 - 1.06)	1	.67	0.91	(0.58 - 1.42)
2	.51	1.06	(0.90 - 1.25)	2	.31	0.80	(0.51 - 1.23)	2	.02 *	0.59	(0.37 - 0.93)	2	.55	0.87	(0.54 - 1.38)
3	.69	1.03	(0.88 - 1.22)	3	.31	0.80	(0.51 - 1.24)	3	.02 *	0.57	(0.36 - 0.91)	3	.46	0.84	(0.52 - 1.34)
**(F) Death due to General Infectious Causes**				**(F) Death due to General Infectious Causes**				**(F) Death due to General Infectious Causes**				**(F) Death due to General Infectious Causes**			
	**Raw Incidence**	**Incidence Rates per 1000 Person-Years**			**Raw Incidence**	**Incidence Rates per 1000 Person-Years**			**Raw Incidence**	**Incidence Rates per 1000 Person-Years**			**Raw Incidence**	**Incidence Rates per 1000 Person-Years**	
Male	726	5.36	(4.98 - 5.77)	Male	97	12.64	(10.26 - 15.40)	Male	71	19.74	(15.45 - 24.84)	Male	104	34.79	(28.51 - 42.00)
Female	421	5.41	(4.91 - 5.95)	Female	75	18.22	(14.36 - 22.78)	Female	57	24.11	(18.31 - 31.13)	Female	49	21.19	(15.72 - 27.92)
**Sequential Cox Regression Analysis**				**Sequential Cox Regression Analysis**				**Sequential Cox Regression Analysis**				**Sequential Cox Regression Analysis**			
**Model**	***P* value**	**aHR**	**95% CI**	**Model**	***P* value**	**aHR**	**95% CI**	**Model**	***P* value**	**aHR**	**95% CI**	**Model**	***P* value**	**aHR**	**95% CI**
1	.66	0.97	(0.86 - 1.10)	1	.04 *	0.73	(0.54 - 0.98)	1	.10	0.74	(0.52 - 1.06)	1	.03 *	1.44	(1.03 - 2.03)
2	.96	1.00	(0.88 - 1.14)	2	.08	0.76	(0.55 - 1.04)	2	.15	0.76	(0.53 - 1.10)	2	.06	1.41	(0.98 - 2.01)
3	.78	0.98	(0.87 - 1.11)	3	.13	0.78	(0.57 - 1.08)	3	.13	0.76	(0.52 - 1.09)	3	.10	1.36	(0.94 - 1.95)
**(G) Death due to Spontaneous Bacterial Peritonitis**				**(G) Death due to Spontaneous Bacterial Peritonitis**				**(G) Death due to Spontaneous Bacterial Peritonitis**				**(G) Death due to Spontaneous Bacterial Peritonitis**			
	**Raw Incidence**	**Incidence Rates per 1000 Person-Years**			**Raw Incidence**	**Incidence Rates per 1000 Person-Years**			**Raw Incidence**	**Incidence Rates per 1000 Person-Years**			**Raw Incidence**	**Incidence Rates per 1000 Person-Years**	
Male	29	0.21	(0.14 - 0.31)	Male	4	0.52	(0.14 - 1.33)	Male	4	1.11	(0.30 - 2.85)	Male	2	0.67	(0.08 - 2.41)
Female	12	0.15	(0.08 - 0.27)	Female	1	0.24	(0.01 - 1.35)	Female	4	1.69	(0.46 - 4.33)	Female	4	1.73	(0.47 - 4.42)
**Sequential Cox Regression Analysis**				**Sequential Cox Regression Analysis**				**Sequential Cox Regression Analysis**				**Sequential Cox Regression Analysis**			
**Model**	***P* value**	**aHR**	**95% CI**	**Model**	***P* value**	**aHR**	**95% CI**	**Model**	***P* value**	**aHR**	**95% CI**	**Model**	***P* value**	**aHR**	**95% CI**
1	.34	1.39	(0.71 - 2.73)	1	.44	2.40	(0.27 - 21.58)	1	.48	0.61	(0.15 - 2.44)	1	.23	0.35	(0.06 - 1.94)
2	.14	1.70	(0.84 - 3.45)	2	.74	1.48	(0.15 - 14.93)	2	.61	0.68	(0.16 - 2.95)	2	.15	0.27	(0.04 - 1.64)

Model	P value	aHR	95% CI	Model	P value	aHR	95% CI	Model	P value	aHR	95% CI	Model	P value	aHR	95% CI
3	.17	1.64	(0.81 - 3.32)	3	.68	1.63	(0.16 - 16.44)	3	.62	0.69	(0.16 - 2.98)	3	.20	0.31	(0.05 - 1.90)

(H) Death due to Gneral Renal Causes

	Raw Incidence	Incidence Rates per 1000 Person-Years			Raw Incidence	Incidence Rates per 1000 Person-Years			Raw Incidence	Incidence Rates per 1000 Person-Years			Raw Incidence	Incidence Rates per 1000 Person-Years	
Male	26	0.19	(0.13 - 0.28)	Male	7	0.91	(0.37 - 1.88)	Male	0	0.00	(0.00 - 1.03)	Male	0	0.00	(0.00 - 1.23)
Female	17	0.22	(0.13 - 0.35)	Female	2	0.49	(0.06 - 1.75)	Female	1	0.42	(0.01 - 2.35)	Female	1	0.43	(0.01 - 2.41)

Sequential Cox Regression Analysis

Model	*P* value	aHR	95% CI	Model	*P* value	aHR	95% CI	Model	*P* value	aHR	95% CI	Model	*P* value	aHR	95% CI
1	.75	0.90	(0.49 - 1.67)	1	.38	2.02	(0.42 - 9.76)	1	1.00	0.00	(0.00 - Inf)	1	1.00	0.00	(0.00 - Inf)
2	.90	0.96	(0.51 - 1.81)	2	.50	1.74	(0.35 - 8.65)	2	1.00	0.00	(0.00 - Inf)	2	1.00	0.00	(0.00 - Inf)
3	.84	0.94	(0.50 - 1.76)	3	.50	1.73	(0.35 - 8.62)	3	1.00	0.00	(0.00 - Inf)	3	1.00	0.00	(0.00 - Inf)

(I) Death due to General Respiratory Cuases

	Raw Incidence	Incidence Rates per 1000 Person-Years			Raw Incidence	Incidence Rates per 1000 Person-Years			Raw Incidence	Incidence Rates per 1000 Person-Years			Raw Incidence	Incidence Rates per 1000 Person-Years	
Male	138	1.02	(0.86 - 1.20)	Male	22	2.87	(1.80 - 4.34)	Male	10	2.78	(1.33 - 5.11)	Male	15	5.02	(2.81 - 8.26)
Female	143	1.84	(1.55 - 2.16)	Female	16	3.89	(2.22 - 6.30)	Female	15	6.35	(3.56 - 10.44)	Female	12	5.19	(2.68 - 9.05)

Sequential Cox Regression Analysis

Model	*P* value	aHR	95% CI	Model	*P* value	aHR	95% CI	Model	*P* value	aHR	95% CI	Model	*P* value	aHR	95% CI
1	< .001 ***	0.55	(0.43 - 0.69)	1	.38	0.75	(0.39 - 1.43)	1	.02 *	0.39	(0.18 - 0.88)	1	.69	0.86	(0.40 - 1.83)
2	< .001 ***	0.59	(0.46 - 0.75)	2	.42	0.75	(0.38 - 1.50)	2	.04 *	0.42	(0.18 - 0.97)	2	.53	1.30	(0.58 - 2.93)
3	< .001 ***	0.58	(0.45 - 0.74)	3	.47	0.77	(0.39 - 1.54)	3	.04 *	0.42	(0.18 - 0.96)	3	.49	1.34	(0.59 - 3.03)

(J) Death due to Sepsis

	Raw Incidence	Incidence Rates per 1000 Person-Years			Raw Incidence	Incidence Rates per 1000 Person-Years			Raw Incidence	Incidence Rates per 1000 Person-Years			Raw Incidence	Incidence Rates per 1000 Person-Years	
Male	477	3.52	(3.22 - 3.85)	Male	67	8.73	(6.77 - 11.08)	Male	49	13.63	(10.10 - 17.98)	Male	74	24.75	(19.49 - 30.98)
Female	264	3.39	(3.00 - 3.83)	Female	56	13.60	(10.29 - 17.63)	Female	40	16.92	(12.12 - 22.97)	Female	28	12.11	(8.06 - 17.46)

Sequential Cox Regression Analysis

Model	*P* value	aHR	95% CI	Model	*P* value	aHR	95% CI	Model	*P* value	aHR	95% CI	Model	*P* value	aHR	95% CI
1	.85	1.01	(0.87 - 1.18)	1	.03 *	0.67	(0.47 - 0.95)	1	.14	0.73	(0.48 - 1.11)	1	.009 **	1.79	(1.16 - 2.76)
2	.79	1.02	(0.87 - 1.19)	2	.08	0.71	(0.49 - 1.04)	2	.28	0.78	(0.50 - 1.22)	2	.02 *	1.73	(1.09 - 2.73)
3	1.00	1.00	(0.86 - 1.17)	3	.11	0.74	(0.51 - 1.07)	3	.26	0.77	(0.50 - 1.21)	3	.03 *	1.65	(1.04 - 2.63)

(J) Death due to Variceal Hemorrhage

	Raw Incidence	Incidence Rates per 1000 Person-Years			Raw Incidence	Incidence Rates per 1000 Person-Years			Raw Incidence	Incidence Rates per 1000 Person-Years			Raw Incidence	Incidence Rates per 1000 Person-Years	
Male	144	1.06	(0.90 - 1.25)	Male	15	1.96	(1.09 - 3.22)	Male	12	3.34	(1.73 - 5.82)	Male	10	3.35	(1.61 - 6.14)
Female	43	0.55	(0.40 - 0.74)	Female	10	2.43	(1.17 - 4.46)	Female	7	2.96	(1.19 - 6.09)	Female	9	3.89	(1.78 - 7.38)

Sequential Cox Regression Analysis

Model	*P* value	aHR	95% CI	Model	*P* value	aHR	95% CI	Model	*P* value	aHR	95% CI	Model	*P* value	aHR	95% CI
1	< .001 ***	1.93	(1.38 - 2.72)	1	.69	0.85	(0.38 - 1.90)	1	.96	0.98	(0.38 - 2.49)	1	.52	0.74	(0.30 - 1.83)
2	< .001 ***	1.87	(1.32 - 2.65)	2	.45	0.72	(0.31 - 1.67)	2	.80	0.88	(0.33 - 2.32)	2	.42	0.68	(0.27 - 1.73)
3	< .001 ***	1.83	(1.29 - 2.60)	3	.39	0.69	(0.30 - 1.60)	3	.71	0.83	(0.32 - 2.19)	3	.30	0.61	(0.23 - 1.56)

* p < 0.05, ** p < 0.01, *** p < 0.001

† FM indicates Final Model

Footnote: *Model 1 includes VOI (variable of interest) and demographics; Model 2 includes Model 1 terms with the addition of comorbidities, and liver disease etiologies; Model 3 includes Model 2 terms with the addition of hepatic variables, MELD score, and liver laboratory markers; Model 4 includes Model 3 terms with the addition of donor demographics

Supplementary Table 3.1. Sequential Cox Proportional Hazards Models Assessing Sex Within Short Stature (Height <168 cm) and Risk of All-Cause Mortality and Liver Transplantation Across Acute-on-Chronic Liver Failure Grades

Without Acute-on-Chronic Liver Failure

(A) All-Cause Mortality

	Raw Incidence	Incidence Rates per 1000 Person-Years	95% CI
Male	925	44.43	(41.67 - 47.31)
Female	2782	43.11	(41.55 - 44.70)

Sequential Cox Regression Analysis

Model	*P* value	aHR	95% CI
1	.97	1.00	(0.93 - 1.08)
2	.42	1.03	(0.95 - 1.12)
3	.70	1.02	(0.94 - 1.10)

(B) Transplantation

	Raw Incidence	Incidence Rates per 1000 Person-Years	95% CI
Male	2894	139.00	(134.33 - 143.77)
Female	8068	125.01	(122.47 - 127.58)

Sequential Cox Regression Analysis

Model	*P* value	aHR	95% CI
1	< .001 ***	1.12	(1.07 - 1.17)
2	< .001 ***	1.18	(1.13 - 1.23)
3	< .001 ***	1.17	(1.12 - 1.22)

Acute-on-Chronic Liver Failure Grade 1

(A) All-Cause Mortality

	Raw Incidence	Incidence Rates per 1000 Person-Years	95% CI
Male	89	69.71	(56.36 - 85.09)
Female	324	95.02	(85.38 - 105.35)

Sequential Cox Regression Analysis

Model	*P* value	aHR	95% CI
1	.01 *	0.74	(0.58 - 0.94)
2	.02 *	0.74	(0.58 - 0.96)
3	.03 *	0.76	(0.59 - 0.98)

(B) Transplantation

	Raw Incidence	Incidence Rates per 1000 Person-Years	95% CI
Male	558	437.08	(409.65 - 464.80)
Female	1594	467.45	(450.59 - 484.37)

Sequential Cox Regression Analysis

Model	*P* value	aHR	95% CI
1	.90	1.01	(0.91 - 1.11)
2	.91	0.99	(0.90 - 1.10)
3	.66	1.02	(0.92 - 1.13)

Acute-on-Chronic Liver Failure Grade 2

(A) All-Cause Mortality

	Raw Incidence	Incidence Rates per 1000 Person-Years	95% CI
Male	69	113.55	(89.43 - 141.50)
Female	242	128.28	(113.51 - 144.21)

Sequential Cox Regression Analysis

Model	*P* value	aHR	95% CI
1	.11	0.79	(0.60 - 1.05)
2	.08	0.78	(0.58 - 1.03)
3	.09	0.78	(0.59 - 1.04)

(B) Transplantation

	Raw Incidence	Incidence Rates per 1000 Person-Years	95% CI
Male	466	766.89	(731.20 - 799.97)
Female	1280	678.48	(656.88 - 699.53)

Sequential Cox Regression Analysis

Model	*P* value	aHR	95% CI
1	.38	1.05	(0.94 - 1.17)
2	.31	1.06	(0.95 - 1.19)
3	.46	1.04	(0.93 - 1.17)

Acute-on-Chronic Liver Failure Grade 3

(A) All-Cause Mortality

	Raw Incidence	Incidence Rates per 1000 Person-Years	95% CI
Male	72	144.85	(115.09 - 178.91)
Female	302	158.93	(142.76 - 176.16)

Sequential Cox Regression Analysis

Model	*P* value	aHR	95% CI
1	.20	0.84	(0.64 - 1.10)
2	.32	0.87	(0.66 - 1.14)
3	.22	0.84	(0.63 - 1.11)

(B) Transplantation

	Raw Incidence	Incidence Rates per 1000 Person-Years	95% CI
Male	324	651.83	(608.14 - 693.70)
Female	886	466.27	(443.64 - 489.00)

Sequential Cox Regression Analysis

Model	*P* value	aHR	95% CI
1	.005 **	1.21	(1.06 - 1.38)
2	.07	1.14	(0.99 - 1.31)
3	.16	1.11	(0.96 - 1.28)

* $p < 0.05$, ** $p < 0.01$, *** $p < 0.001$

† FM indicates Final Model

Footnote: *Model 1 includes VOI (variable of interest) and demographics; Model 2 includes Model 1 terms with the addition of comorbidities, and liver disease etiologies; Model 3 includes Model 2 terms with the addition of hepatic variables, MELD score, and liver laboratory markers; Model 4 includes Model 3 terms with the addition of donor demographics

Supplementary Table 3.2. Sequential Cox Proportional Hazards Models Assessing Sex Within Intermediate Height (167.64–177.8 cm) and Risk of All-Cause Mortality and Liver Transplantation Across Acute-on-Chronic Liver Failure Grades

Without Acute-on-Chronic Liver Failure

(A) All-Cause Mortality

	Raw Incidence	Incidence Rates per 1000 Person-Years	
Male	2479	40.86	(39.30 - 42.47)
Female	476	38.46	(35.14 - 42.00)

Sequential Cox Regression Analysis

Model	*P* value	aHR	95% CI
1	.66	1.02	(0.93 - 1.13)
2	.78	1.01	(0.92 - 1.12)
3	.91	1.01	(0.91 - 1.11)

(B) Transplantation

	Raw Incidence	Incidence Rates per 1000 Person-Years	
Male	9492	156.46	(153.58 - 159.37)
Female	1811	146.33	(140.14 - 152.68)

Sequential Cox Regression Analysis

Model	*P* value	aHR	95% CI
1	.008 **	1.07	(1.02 - 1.13)
2	< .001 ***	1.12	(1.07 - 1.18)
3	< .001 ***	1.12	(1.07 - 1.18)

Acute-on-Chronic Liver Failure Grade 1

(A) All-Cause Mortality

	Raw Incidence	Incidence Rates per 1000 Person-Years	
Male	267	75.92	(67.38 - 85.17)
Female	74	112.13	(89.08 - 138.71)

Sequential Cox Regression Analysis

Model	*P* value	aHR	95% CI
1	.03 *	0.74	(0.57 - 0.96)
2	.03 *	0.74	(0.56 - 0.97)
3	.02 *	0.72	(0.55 - 0.95)

(B) Transplantation

	Raw Incidence	Incidence Rates per 1000 Person-Years	
Male	1734	493.03	(476.37 - 509.69)
Female	372	563.66	(524.85 - 601.90)

Sequential Cox Regression Analysis

Model	*P* value	aHR	95% CI
1	.69	1.02	(0.91 - 1.15)
2	.78	1.02	(0.90 - 1.14)
3	.59	1.03	(0.92 - 1.16)

Acute-on-Chronic Liver Failure Grade 2

(A) All-Cause Mortality

	Raw Incidence	Incidence Rates per 1000 Person-Years	
Male	200	135.91	(118.80 - 154.49)
Female	45	101.15	(74.74 - 133.00)

Sequential Cox Regression Analysis

Model	*P* value	aHR	95% CI
1	.41	1.15	(0.83 - 1.59)
2	.62	1.09	(0.78 - 1.52)
3	.49	1.13	(0.80 - 1.57)

(B) Transplantation

	Raw Incidence	Incidence Rates per 1000 Person-Years	
Male	1328	902.44	(886.13 - 917.13)
Female	311	699.07	(654.10 - 741.37)

Sequential Cox Regression Analysis

Model	*P* value	aHR	95% CI
1	.56	1.04	(0.92 - 1.18)
2	.37	1.06	(0.93 - 1.21)
3	.31	1.07	(0.94 - 1.21)

Acute-on-Chronic Liver Failure Grade 3

(A) All-Cause Mortality

	Raw Incidence	Incidence Rates per 1000 Person-Years	
Male	243	175.80	(156.08 - 196.91)
Female	59	151.74	(117.56 - 191.33)

Sequential Cox Regression Analysis

Model	*P* value	aHR	95% CI
1	.32	1.16	(0.87 - 1.55)
2	.40	1.14	(0.84 - 1.55)
3	.73	1.06	(0.77 - 1.44)

(B) Transplantation

	Raw Incidence	Incidence Rates per 1000 Person-Years	
Male	851	615.67	(589.44 - 641.40)
Female	222	570.96	(520.09 - 620.73)

Sequential Cox Regression Analysis

Model	*P* value	aHR	95% CI
1	.27	1.09	(0.94 - 1.27)
2	.06	1.16	(0.99 - 1.36)
3	.15	1.12	(0.96 - 1.32)

* $p < 0.05$, ** $p < 0.01$, *** $p < 0.001$

† FM indicates Final Model

Footnote: *Model 1 includes VOI (variable of interest) and demographics; Model 2 includes Model 1 terms with the addition of comorbidities, and liver disease etiologies; Model 3 includes Model 2 terms with the addition of hepatic variables, MELD score, and liver laboratory markers; Model 4 includes Model 3 terms with the addition of donor demographics

Supplementary Table 3.3. Sequential Cox Proportional Hazards Models Assessing Sex Within Tall Stature (>177.8 cm) and Risk of All-Cause Mortality and Liver Transplantation Across Acute-on-Chronic Liver Failure Grades

Without Acute-on-Chronic Liver Failure

(A) All-Cause Mortality

	Raw Incidence	Incidence Rates per 1000 Person-Years	
Male	2109	39.15	(37.53 - 40.82)
Female	37	41.61	(29.46 - 56.90)

Sequential Cox Regression Analysis

Model	*P* value	aHR	95% CI
1	.36	0.86	(0.62 - 1.19)
2	.36	0.86	(0.62 - 1.19)
3	.38	0.86	(0.62 - 1.20)

(B) Transplantation

	Raw Incidence	Incidence Rates per 1000 Person-Years	
Male	8873	164.72	(161.59 - 167.88)
Female	146	164.18	(140.42 - 190.19)

Sequential Cox Regression Analysis

Model	*P* value	aHR	95% CI
1	.58	0.95	(0.81 - 1.12)
2	.95	1.01	(0.85 - 1.19)
3	.69	1.03	(0.88 - 1.22)

Acute-on-Chronic Liver Failure Grade 1

(A) All-Cause Mortality

	Raw Incidence	Incidence Rates per 1000 Person-Years	
Male	260	90.32	(80.09 - 101.38)
Female	5	106.04	(35.34 - 230.35)

Sequential Cox Regression Analysis

Model	*P* value	aHR	95% CI
1	.77	0.87	(0.36 - 2.13)
2	.67	0.82	(0.34 - 2.02)
3	.81	0.90	(0.37 - 2.21)

(B) Transplantation

	Raw Incidence	Incidence Rates per 1000 Person-Years	
Male	1564	543.29	(524.89 - 561.61)
Female	27	572.59	(420.22 - 715.52)

Sequential Cox Regression Analysis

Model	*P* value	aHR	95% CI
1	.65	1.09	(0.75 - 1.60)
2	.82	1.05	(0.71 - 1.54)
3	.69	1.08	(0.74 - 1.59)

Acute-on-Chronic Liver Failure Grade 2

(A) All-Cause Mortality

	Raw Incidence	Incidence Rates per 1000 Person-Years	
Male	170	112.08	(96.64 - 129.04)
Female	3	92.29	(19.45 - 246.68)

Sequential Cox Regression Analysis

Model	*P* value	aHR	95% CI
1	.83	1.14	(0.36 - 3.57)
2	.99	1.01	(0.32 - 3.21)
3	.89	0.92	(0.29 - 2.94)

(B) Transplantation

	Raw Incidence	Incidence Rates per 1000 Person-Years	
Male	1302	858.41	(839.84 - 875.57)
Female	23	707.56	(522.39 - 853.15)

Sequential Cox Regression Analysis

Model	*P* value	aHR	95% CI
1	.39	1.20	(0.79 - 1.82)
2	.37	1.21	(0.80 - 1.84)
3	.49	1.16	(0.76 - 1.76)

Acute-on-Chronic Liver Failure Grade 3

(A) All-Cause Mortality

	Raw Incidence	Incidence Rates per 1000 Person-Years	
Male	225	202.67	(179.39 - 227.54)
Female	1	43.22	(1.09 - 218.29)

Sequential Cox Regression Analysis

Model	*P* value	aHR	95% CI
1	.08	5.83	(0.81 - 41.80)
2	.08	5.79	(0.81 - 41.70)
3	.09	5.53	(0.77 - 39.81)

(B) Transplantation

	Raw Incidence	Incidence Rates per 1000 Person-Years	
Male	842	758.44	(732.15 - 783.36)
Female	27	1166.8<br>0	(NaN - NaN)

Sequential Cox Regression Analysis

Model	*P* value	aHR	95% CI
1	.19	0.77	(0.52 - 1.13)
2	.18	0.77	(0.52 - 1.14)
3	.12	0.73	(0.49 - 1.09)

* p < 0.05, ** p < 0.01, *** p < 0.001

† FM indicates Final Model

Footnote: *Model 1 includes VOI (variable of interest) and demographics; Model 2 includes Model 1 terms with the addition of comorbidities, and liver disease etiologies; Model 3 includes Model 2 terms with the addition of hepatic variables, MELD score, and liver laboratory markers; Model 4 includes Model 3 terms with the addition of donor demographics

Supplementary Table 3.4. Sequential Cox Proportional Hazards Models Assessing Sex Within Lower Body Weight (<77.11 kg) and Risk of All-Cause Mortality and Liver Transplantation Across Acute-on-Chronic Liver Failure Grades

Without Acute-on-Chronic Liver Failure

(A) All-Cause Mortality

	Raw Incidence	Incidence Rates per 1000 Person-Years	
Male	1368	41.85	(39.70 - 44.08)
Female	1822	39.54	(37.78 - 41.36)

Sequential Cox Regression Analysis

Model	*P* value	aHR	95% CI
1	.13	1.06	(0.98 - 1.14)
2	.05	1.08	(1.00 - 1.16)
3	.20	1.05	(0.97 - 1.13)

(B) Transplantation

	Raw Incidence	Incidence Rates per 1000 Person-Years	
Male	4375	133.84	(130.17 - 137.58)
Female	5396	117.10	(114.18 - 120.07)

Sequential Cox Regression Analysis

Model	*P* value	aHR	95% CI
1	< .001 ***	1.15	(1.10 - 1.20)
2	< .001 ***	1.19	(1.14 - 1.24)
3	< .001 ***	1.17	(1.12 - 1.22)

Acute-on-Chronic Liver Failure Grade 1

(A) All-Cause Mortality

	Raw Incidence	Incidence Rates per 1000 Person-Years	
Male	179	87.45	(75.56 - 100.53)
Female	223	88.97	(78.11 - 100.80)

Sequential Cox Regression Analysis

Model	*P* value	aHR	95% CI
1	.94	1.01	(0.82 - 1.23)
2	.97	1.00	(0.81 - 1.23)
3	.98	1.00	(0.81 - 1.23)

(B) Transplantation

	Raw Incidence	Incidence Rates per 1000 Person-Years	
Male	893	436.25	(414.62 - 458.05)
Female	1067	425.69	(406.23 - 445.32)

Sequential Cox Regression Analysis

Model	*P* value	aHR	95% CI
1	.04 *	1.10	(1.01 - 1.21)
2	.13	1.08	(0.98 - 1.18)
3	.07	1.09	(0.99 - 1.20)

Acute-on-Chronic Liver Failure Grade 2

(A) All-Cause Mortality

	Raw Incidence	Incidence Rates per 1000 Person-Years	
Male	114	120.18	(100.17 - 142.59)
Female	150	113.15	(96.60 - 131.45)

Sequential Cox Regression Analysis

Model	*P* value	aHR	95% CI
1	.87	1.02	(0.79 - 1.32)
2	.94	0.99	(0.76 - 1.29)
3	.95	1.01	(0.77 - 1.31)

(B) Transplantation

	Raw Incidence	Incidence Rates per 1000 Person-Years	
Male	703	741.13	(712.01 - 768.75)
Female	804	606.50	(579.61 - 632.92)

Sequential Cox Regression Analysis

Model	*P* value	aHR	95% CI
1	.29	1.06	(0.95 - 1.18)
2	.27	1.06	(0.95 - 1.19)
3	.65	1.03	(0.92 - 1.14)

Acute-on-Chronic Liver Failure Grade 3

(A) All-Cause Mortality

	Raw Incidence	Incidence Rates per 1000 Person-Years	
Male	127	193.36	(163.83 - 225.67)
Female	189	152.28	(132.73 - 173.49)

Sequential Cox Regression Analysis

Model	*P* value	aHR	95% CI
1	.50	1.08	(0.86 - 1.37)
2	.66	1.06	(0.82 - 1.36)
3	.82	1.03	(0.80 - 1.33)

(B) Transplantation

	Raw Incidence	Incidence Rates per 1000 Person-Years	
Male	444	675.99	(638.70 - 711.68)
Female	536	431.86	(404.09 - 459.96)

Sequential Cox Regression Analysis

Model	*P* value	aHR	95% CI
1	< .001 ***	1.29	(1.13 - 1.47)
2	.002 **	1.25	(1.09 - 1.44)
3	.02 *	1.19	(1.03 - 1.37)

* p < 0.05, ** p < 0.01, *** p < 0.001

† FM indicates Final Model

Footnote: *Model 1 includes VOI (variable of interest) and demographics; Model 2 includes Model 1 terms with the addition of comorbidities, and liver disease etiologies; Model 3 includes Model 2 terms with the addition of hepatic variables, MELD score, and liver laboratory markers; Model 4 includes Model 3 terms with the addition of donor demographics

Supplementary Table 3.5. Sequential Cox Proportional Hazards Models Assessing Sex Within Intermediate Body Weight (77.11–93.89 kg) and Risk of All-Cause Mortality and Liver Transplantation Across Acute-on-Chronic Liver Failure Grades

(A) All-Cause Mortality

	Without Acute-on-Chronic Liver Failure				Acute-on-Chronic Liver Failure Grade 1				Acute-on-Chronic Liver Failure Grade 2				Acute-on-Chronic Liver Failure Grade 3			
	Raw Incidence	Incidence Rates per 1000 Person-Years			Raw Incidence	Incidence Rates per 1000 Person-Years			Raw Incidence	Incidence Rates per 1000 Person-Years			Raw Incidence	Incidence Rates per 1000 Person-Years		
Male	1953	39.38	(37.69 - 41.13)		184	68.01	(58.81 - 78.16)		135	118.71	(100.48 - 138.94)		180	175.79	(152.95 - 200.52)	
Female	912	43.42	(40.70 - 46.26)		90	91.04	(73.84 - 110.72)		80	168.92	(136.27 - 205.76)		88	142.65	(116.00 - 172.76)	

Sequential Cox Regression Analysis

Model	*P* value	aHR	95% CI	*P* value	aHR	95% CI	*P* value	aHR	95% CI	*P* value	aHR	95% CI
	**Without Acute-on-Chronic Liver Failure**			**Acute-on-Chronic Liver Failure Grade 1**			**Acute-on-Chronic Liver Failure Grade 2**			**Acute-on-Chronic Liver Failure Grade 3**		
1	.15	0.93	(0.84 - 1.03)	.02 *	0.69	(0.50 - 0.95)	.15	0.77	(0.54 - 1.10)	.99	1.00	(0.73 - 1.37)
2	.09	0.92	(0.83 - 1.01)	.05	0.72	(0.52 - 1.00)	.10	0.74	(0.51 - 1.06)	.92	1.02	(0.73 - 1.41)
3	.06	0.91	(0.82 - 1.00)	.06	0.72	(0.52 - 1.01)	.11	0.74	(0.52 - 1.07)	.88	0.97	(0.70 - 1.35)

(B) Transplantation

	Without Acute-on-Chronic Liver Failure				Acute-on-Chronic Liver Failure Grade 1				Acute-on-Chronic Liver Failure Grade 2				Acute-on-Chronic Liver Failure Grade 3			
	Raw Incidence	Incidence Rates per 1000 Person-Years			Raw Incidence	Incidence Rates per 1000 Person-Years			Raw Incidence	Incidence Rates per 1000 Person-Years			Raw Incidence	Incidence Rates per 1000 Person-Years		
Male	7709	155.45	(152.27 - 158.67)		1360	502.68	(483.66 - 521.69)		1023	899.58	(880.62 - 916.44)		630	615.28	(584.70 - 645.20)	
Female	2854	135.87	(131.26 - 140.58)		553	559.39	(527.80 - 590.63)		447	943.81	(919.09 - 962.77)		327	530.06	(489.80 - 570.03)	

Sequential Cox Regression Analysis

Model	*P* value	aHR	95% CI	*P* value	aHR	95% CI	*P* value	aHR	95% CI	*P* value	aHR	95% CI
	**Without Acute-on-Chronic Liver Failure**			**Acute-on-Chronic Liver Failure Grade 1**			**Acute-on-Chronic Liver Failure Grade 2**			**Acute-on-Chronic Liver Failure Grade 3**		
1	< .001 ***	1.14	(1.08 - 1.20)	.90	0.99	(0.88 - 1.12)	.48	1.05	(0.91 - 1.21)	.57	0.95	(0.81 - 1.12)
2	< .001 ***	1.20	(1.13 - 1.26)	.78	0.98	(0.87 - 1.11)	.32	1.08	(0.93 - 1.25)	.43	0.93	(0.79 - 1.11)
3	< .001 ***	1.18	(1.12 - 1.25)	.84	1.01	(0.89 - 1.15)	.30	1.08	(0.93 - 1.25)	.32	0.92	(0.77 - 1.09)

* $p < 0.05$, ** $p < 0.01$, *** $p < 0.001$

† FM indicates Final Model

Footnote: *Model 1 includes VOI (variable of interest) and demographics; Model 2 includes Model 1 terms with the addition of comorbidities, and liver disease etiologies; Model 3 includes Model 2 terms with the addition of hepatic variables, MELD score, and liver laboratory markers; Model 4 includes Model 3 terms with the addition of donor demographics

Supplementary Table 3.6. Sequential Cox Proportional Hazards Models Assessing Sex Within Higher Body Weight (>93.89 kg) and Risk of All-Cause Mortality and Liver Transplantation Across Acute-on-Chronic Liver Failure Grades

Without Acute-on-Chronic Liver Failure

(A) All-Cause Mortality

	Raw Incidence	Incidence Rates per 1000 Person-Years	
Male	2192	41.30	(39.62 - 43.03)
Female	561	52.34	(48.20 - 56.72)

Sequential Cox Regression Analysis

Model	*P* value	aHR	95% CI
1	.06	0.91	(0.82 - 1.00)
2	.11	0.92	(0.83 - 1.02)
3	.11	0.92	(0.83 - 1.02)

(B) Transplantation

	Raw Incidence	Incidence Rates per 1000 Person-Years	
Male	9175	172.87	(169.66 - 176.11)
Female	1775	165.60	(158.60 - 172.77)

Sequential Cox Regression Analysis

Model	*P* value	aHR	95% CI
1	.02 *	1.07	(1.01 - 1.13)
2	< .001 ***	1.12	(1.06 - 1.19)
3	< .001 ***	1.14	(1.08 - 1.21)

Acute-on-Chronic Liver Failure Grade 1

(A) All-Cause Mortality

	Raw Incidence	Incidence Rates per 1000 Person-Years	
Male	253	86.65	(76.69 - 97.44)
Female	90	144.70	(117.99 - 174.83)

Sequential Cox Regression Analysis

Model	*P* value	aHR	95% CI
1	.001 **	0.64	(0.49 - 0.84)
2	.002 **	0.63	(0.47 - 0.84)
3	.002 **	0.64	(0.48 - 0.85)

(B) Transplantation

	Raw Incidence	Incidence Rates per 1000 Person-Years	
Male	1603	548.99	(530.73 - 567.15)
Female	373	599.69	(559.98 - 638.44)

Sequential Cox Regression Analysis

Model	*P* value	aHR	95% CI
1	.97	1.00	(0.89 - 1.13)
2	.80	0.98	(0.86 - 1.12)
3	.68	0.97	(0.86 - 1.11)

Acute-on-Chronic Liver Failure Grade 2

(A) All-Cause Mortality

	Raw Incidence	Incidence Rates per 1000 Person-Years	
Male	190	125.81	(109.49 - 143.60)
Female	60	106.25	(82.07 - 134.65)

Sequential Cox Regression Analysis

Model	*P* value	aHR	95% CI
1	.77	1.05	(0.76 - 1.44)
2	.76	0.95	(0.69 - 1.32)
3	.71	0.94	(0.68 - 1.31)

(B) Transplantation

	Raw Incidence	Incidence Rates per 1000 Person-Years	
Male	1370	907.15	(891.38 - 921.31)
Female	363	642.83	(601.75 - 682.40)

Sequential Cox Regression Analysis

Model	*P* value	aHR	95% CI
1	.32	1.07	(0.94 - 1.21)
2	.18	1.10	(0.96 - 1.25)
3	.19	1.09	(0.96 - 1.25)

Acute-on-Chronic Liver Failure Grade 3

(A) All-Cause Mortality

	Raw Incidence	Incidence Rates per 1000 Person-Years	
Male	233	178.03	(157.67 - 199.86)
Female	85	187.17	(152.33 - 226.15)

Sequential Cox Regression Analysis

Model	*P* value	aHR	95% CI
1	.97	1.01	(0.77 - 1.32)
2	.75	1.05	(0.78 - 1.40)
3	.71	0.95	(0.71 - 1.27)

(B) Transplantation

	Raw Incidence	Incidence Rates per 1000 Person-Years	
Male	943	720.54	(695.37 - 744.72)
Female	272	598.95	(552.25 - 644.36)

Sequential Cox Regression Analysis

Model	*P* value	aHR	95% CI
1	.04 *	1.17	(1.01 - 1.36)
2	.07	1.15	(0.99 - 1.35)
3	.24	1.10	(0.94 - 1.29)

* p < 0.05, ** p < 0.01, *** p < 0.001

† FM indicates Final Model

Footnote: *Model 1 includes VOI (variable of interest) and demographics; Model 2 includes Model 1 terms with the addition of comorbidities, and liver disease etiologies; Model 3 includes Model 2 terms with the addition of hepatic variables, MELD score, and liver laboratory markers; Model 4 includes Model 3 terms with the addition of donor demographics
