# Supplementary material for: The Clinical Implications of Sex on Waitlist Outcomes in Patients With Acute-on-Chronic Liver Failure
Source: Gastro Hep Adv. 2026 Apr 13;5(7):100970. doi: 10.1016/j.gastha.2026.100970 (PMC13207543; doi:10.1016/j.gastha.2026.100970)
Supplement: Supplementary file 9 [file mmc9.pdf]

Supplementary Tables

Supplementary Table 1 presents sensitivity analyses evaluating sex and waitlist outcomes across ACLF grades using event-specific Cox proportional hazards models. In the fully adjusted model (model 4), there was comparable all-cause mortality in ACLF grades 1-3 and a higher transplant rate across ACLF grades. Supplementary Table 2 presents additional model outputs for waitlist outcomes and cause-specific mortality endpoints across ACLF grades, including adjusted estimates by sequential models where applicable; statistically significant associations were present for selected outcomes and strata, while many estimates were not statistically significant.

Supplementary Tables 3.1–3.3 report sequential Cox models stratified by height (short, intermediate, tall). In the fully adjusted model (model 4), male sex was associated with lower all-cause mortality in ACLF grade 1 in the short stratum (adjusted hazard ratio [aHR]: 0.76; 95% CI: 0.59–0.98; $P = .03$) and in the intermediate stratum (aHR: 0.73; 95% CI: 0.55–0.96; $P = .02$).

For transplantation rate in model 4, male sex was associated with higher transplantation rates in the no-ACLF stratum in the short height group (aHR: 1.17; 95% CI: 1.12–1.22; $P < .001$) and in the intermediate height group (aHR: 1.12; 95% CI: 1.07–1.18; $P < .001$).

Supplementary Tables 3.4–3.6 report sequential Cox models stratified by body weight (low, intermediate, higher). In model 4, male sex was associated with lower all-cause mortality in ACLF grade 1 in the intermediate weight stratum (aHR: 0.71; 95% CI: 0.51–0.99; $P = .05$) and

in the higher weight stratum (aHR: 0.64; 95% CI: 0.48–0.85; $P = .002$). For transplantation rate, male sex was associated with higher transplantation rates in the no-ACLF stratum in the low, intermediate, and higher weight strata (model 4 aHR: 1.17, 1.18, and 1.14, respectively; all $P < .001$). In the low weight stratum, male sex was also associated with higher transplantation in ACLF grade 3 (model 4 aHR: 1.19; 95% CI: 1.03–1.37; $P = .02$).
